# Global Burden and Temporal Trends of Early‐Onset Tracheal, Bronchial, and Lung Cancer: An Analysis of GLOBOCAN 2022 and GBD 2023 Data

**DOI:** 10.1002/advs.77778

**Published:** 2026-09-16

**Authors:** Ye Tian, Weihao Zhang, Yao Lu, Bruce F. O'Hara, Jingjing Hu, Hanqi Feng, Jing Di, Lei Zhang, Xueying Yang

**Affiliations:** ^1^ Department of Thoracic Surgery The Fourth Affiliated Hospital of China Medical University Shenyang China; ^2^ Department of Biology University of Kentucky Lexington Kentucky USA; ^3^ Department of Pathology Division of Anatomic Pathology University of California San Diego Health La Jolla California USA; ^4^ Machine Learning Department School of Computer Science Carnegie Mellon University Pittsburgh Pennsylvania USA

**Keywords:** disability‐adjusted life years, early‐onset tracheal, bronchial, and lung cancer, human development index, incidence, mortality

## Abstract

**Background** Early‐onset tracheal, bronchial, and lung (EOTBL) cancer represents a distinct clinical entity. This study aimed to assess the global burden and temporal trends of EOTBL cancer, focusing on socioeconomic disparities, to inform global cancer control strategies.

**Methods** Using GLOBOCAN 2022 and GBD 2023 data, we analyzed EOTBL cancer incidence, mortality, and attributable risk factors across distinct geographic regions and Human Development Index (HDI) tiers.

**Findings** In 2022, EOTBL cancer caused 137,705 incident cases and 72,646 deaths globally. Eastern Asia showed the highest incidence. Socioeconomically, regions with a High HDI presented the highest incidence burden. However, the Medium‐ and Low‐HDI regions experienced disproportionately high mortality burden. The temporal trends in EOTBL cancer exhibited profound national heterogeneity, closely tied to geography, human development, and sex. Risk profiles varied drastically: active smoking remained the primary driver in countries like Russia, whereas ambient particulate matter pollution emerged as a major determinant in rapidly developing economies like China.

**Interpretation** EOTBL cancer pattern exhibits notable disparities across sex, geographic regions, and HDI levels. Framing effective global policies requires moving beyond generalized interventions toward tailored, region‐specific public health strategies.

## Graphical abstract

This study systematically assessed the global burden and temporal trends of EOTBL cancer in individuals aged 15–49 years. Smoking, ambient particulate matter pollution, and secondhand smoke remain dominant risk factors. Countries within the same Human Development Index (HDI) category exhibit distinct epidemiological trajectories, underscoring the need for tailored, context‐specific prevention and screening strategies.

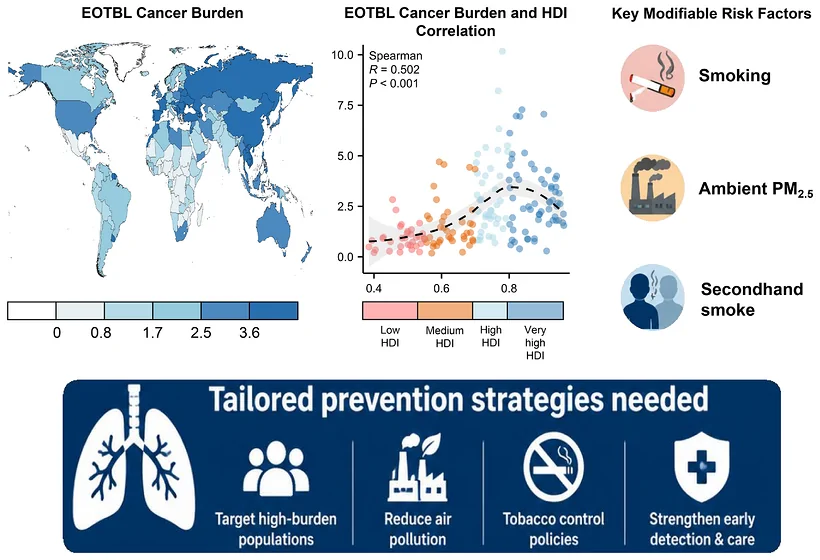

## 1 Background

In 2022, tracheal, bronchial, and lung (TBL) cancer remained the leading cause of global cancer incidence and mortality. It accounted for approximately 2.5 million new cases (12.4% of all cancer cases) and an estimated 1.8 million deaths (18.7% of all cancer deaths) [1]. Additionally, TBL cancer imposed a substantial economic burden, costing up to 3.9 trillion US dollars in 2017, the highest among all cancer types [2]. Although TBL cancer primarily affects individuals aged 65 years or older, accounting for 83% of all cases [3], the global trend in early‐onset TBL (EOTBL) cancer (identified as occurring in adults under 50 years of age) presents an unclear picture, with incidence patterns varying considerably across recent epidemiological studies [4, 5, 6]. Particularly in Eastern Asia, EOTBL is characterized by a predominance of non‐smokers, females, and adenocarcinoma histological subtypes, with the vast majority of patients harboring driver mutations in *EGFR*, *ERBB2*, or *ALK* [7]. Compared with late‐onset disease, EOTBL cancer has been less studied. However, the survival and prognosis of younger patients remain controversial. Some studies have reported that survival is similar between younger and older individuals, whereas other studies have shown that younger patients have a worse prognosis [7, 8, 9]. These studies have not yet provided a systematic and comprehensive assessment of the global burden of EOTBL or its preventive strategies despite revealing its heterogeneous distribution across regions [10, 11]. Additionally, young patients with EOTBL cancer face unique age‐specific challenges, including concerns regarding fertility preservation, financial hardship, and psychosocial distress. Several studies have indicated that patients with early‐onset cancer experience cancer‐related financial difficulties more frequently than their elderly counterparts [12, 13, 14]. Therefore, understanding the latest EOTBL epidemiological patterns and evolving trends, including risk factors, is crucial to reduce the overall burden and develop prevention strategies for this type of cancer.

The Global Cancer Observatory (GLOBOCAN) 2022 and Global Burden of Disease (GBD) 2023 studies are comprehensive research initiatives that provide critical insights into the global health landscape. By leveraging these updated datasets, this study assesses the global and country‐specific burden of EOTBL cancer. It also presents a detailed trajectory of the incidence and mortality patterns of this cancer subtype across a globally representative selection of countries spanning six continents. These findings provide a scientific basis for developing targeted prevention and control strategies for EOTBL cancer across diverse populations.

## 2 Methods

### 2.1 Study Design

Since the incidence of TBL cancer in people under 15 years of age is rare, this study defined patients aged 15–49 years at the time of TBL cancer diagnosis as having EOTBL cancer, which is consistent with previous studies [15]. TBL cancer cases were classified according to the coding criteria of the International Classification of Diseases, 10th Revision (ICD‐10). Among them, TBL cancer is coded as C33 or C34. Ethical approval and informed consent were not required for this study because it was based on publicly available, anonymized data aggregated at the population level.

### 2.2 Data Source

This study evaluated the burden of EOTBL cancer (15–49 years) in compliance with the Guidelines for Accurate and Transparent Health Estimates Reporting (GATHER) [16]. We comprehensively utilized two primary data sources to leverage their complementary methodological strengths. GLOBOCAN 2022 was employed to capture the most authoritative cross‐sectional snapshot of contemporary global and regional disease burdens, whereas GBD 2023 was used to trace long‐term temporal trends and assess risk‐attributable burdens across key representative countries worldwide. Importantly, the analyses utilizing these datasets were conducted within strictly defined boundaries without data crossover.

Data from 185 countries and territories were stratified into four Human Development Index (HDI) tiers using 2023 United Nations data (Table S2), and grouped into 19 epidemiological regions (with Melanesia, Micronesia, and Polynesia consolidated as the “Pacific Islands”). GLOBOCAN age‐standardized rates (ASRs), specifically the age‐standardized incidence rate (ASIR) and age‐standardized mortality rate (ASMR), were calculated using the World Standard Population.

Based on the GBD 2023 data, we extracted age‐ and sex‐specific incidence, mortality, and disability‐adjusted life years (DALY) rates for a purposely curated panel of 16 representative countries. To ensure robust global representation, this cohort encompassed both the major nations driving the absolute global burden and strategically selected countries stratified across the four HDI tiers. This selection purposefully captured the highest disease burden, divergent epidemiological patterns, and contrasting policy‐relevant contexts.

Regarding the risk‐attributable burden, we initially analyzed 16 GBD‐defined risk factors for EOTBL. To establish rigorous selection criteria, these factors were ranked based on their relative contributions to national and global Population Attributable Fractions (PAFs). Active smoking, secondhand smoke, ambient particulate matter pollution, and household air pollution from solid fuels were selected for detailed presentation because they were consistently among the leading individual risk‐specific contributors across the examined settings. Crucially, individual PAFs reflect the fraction of DALYs averted if a specific risk is reduced to the theoretical minimum risk exposure level (TMREL). Because GBD models account for multicausal biological realities with overlapping exposures, these independent PAFs are not mutually exclusive, and their simple sum can exceed 100%.

### 2.3 Country Selection

To minimize subjective selection bias, we applied a prespecified, transparent, criteria‐based framework for country selection. We first established a primary panel of eight populous countries (China, India, the USA, Brazil, Russia, Nigeria, Germany, and Australia) based on four concurrent criteria: (1) geographic diversity, representing all six inhabited continents; (2) socioeconomic stratification, spanning all four UN‐defined HDI tiers (Very High: USA, Germany, Australia, and Russia; High: China and Brazil; Medium: India; Low: Nigeria); (3) demographic weight, collectively capturing approximately 50% of the global population; and (4) health‐system heterogeneity, reflecting diverse cancer‐control contexts. To mitigate the limitations of prioritizing demographic size alone, we further established a supplementary panel of eight countries using complementary prespecified criteria. These countries were selected to capture epidemiological and policy‐relevant contexts that were not fully represented in the primary population‐based panel, including countries with high region‐specific EOTBL burden, divergent temporal trends, distinct exposure profiles, and diverse cancer‐control policy environments across HDI strata. The country‐specific rationale for inclusion is provided in Table S1.

### 2.4 Statistical Analysis

Estimates of absolute numbers, proportions, ASIR, and ASMR for EOTBL cancer were reported for the selected countries based on GBD 2023. Truncated ASRs per 100 000 person‐years were calculated with standardized adjustments for age, sex, and geographic location to account for methodological variations in measurement [17]. To ensure cross‐national comparability, age standardization was performed using the World Standard Population, as proposed by Segi and later modified by Doll et al. [18]. Differences across sexes and nations were further examined to illustrate key epidemiological patterns.

Temporal trends in incidence and mortality were evaluated using Joinpoint regression analysis [19]. This method employs a logarithmic linear model to identify significant changes in disease burden over time. We utilized the grid search method to determine the optimal joinpoints by minimizing mean squared errors (MSE), followed by Monte Carlo permutation tests to select the final model (ranging from 0 to 5 joinpoints). The overall trend was quantified using the average annual percentage change (AAPC) with a corresponding 95% confidence interval (CI). AAPC was significantly greater than zero (*p* < 0.05), indicating an increasing trend during the study period.

Furthermore, the associations between country‐level ASRs or mortality‐to‐incidence ratios (MIRs) and the HDI were visualized using scatter plots and assessed using Spearman's correlation analysis. All statistical computations and visualizations were performed using R software (version 4.3.3, R Foundation for Statistical Computing, Vienna, Austria).

## 3 Results

### 3.1 Burden of EOTBL Cancer in 2022

**FIGURE 1:**
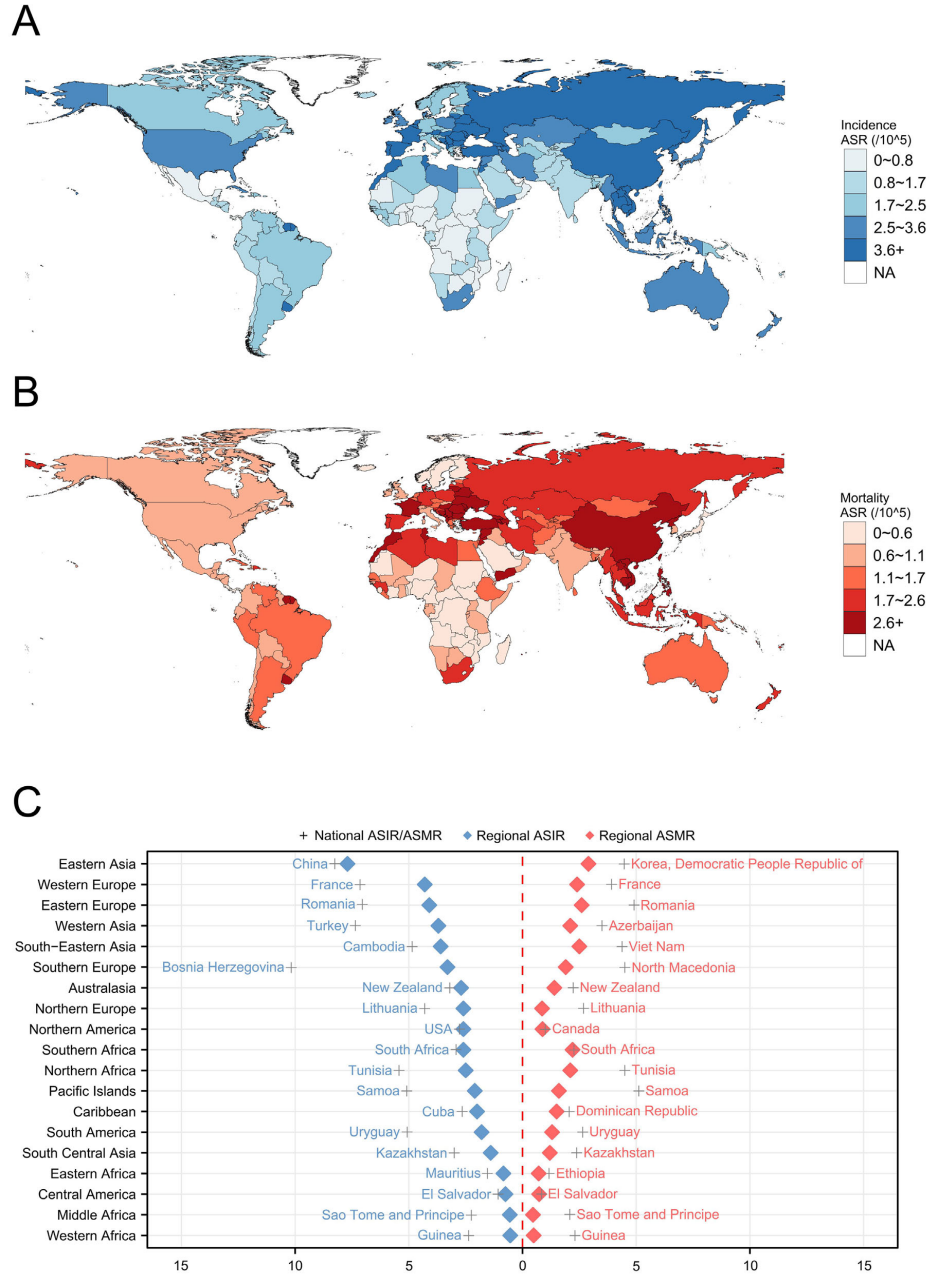
Global and national burden of EOTBL cancers in 2022. (A, B) Global maps illustrating age‐standardized incidence (A) and mortality (B) rates across different countries. (C) Age‐standardized incidence and mortality rates by UN region, highlighting the specific countries with the highest rates within each region.

In 2022, an estimated 137 705 incident cases and 72 646 cancer‐related deaths from EOTBL cancer were recorded worldwide, corresponding to an ASIR of 3.4 per 100 000 and an ASMR of 1.8 per 100 000 (Table 1). Regionally, Eastern Asia carried the heaviest burden of EOTBL cancer globally in terms of both incidence and mortality. This region recorded 71 277 incident cases and 27 234 deaths attributable to this disease. Correspondingly, it reported the highest ASIR (7.7) and ASMR (2.9) values worldwide. At the national level, the burden of EOTBL cancer varied substantially across the 185 countries and territories (Figure 1A,B, Table S3). The highest ASIR burden clusters were in Eastern Asia, Western Europe, and Eastern Europe, as exemplified by China (8.21), France (7.06), and Romania (6.97), whereas the lowest ASIRs were concentrated in Sub‐Saharan Africa, including Niger (0.21), Nigeria (0.24), and Mozambique (0.28). Similar concentrations were observed for ASMR, with the highest rates in regions like Eastern Asia and Eastern Europe and countries such as the Democratic People's Republic of Korea (4.53) and Romania (4.90), and the lowest in countries such as Norway (0.10) and Sweden (0.25) (Figure 1C).

**TABLE 1:** Estimated absolute numbers, proportions, and ASRs of incidence and mortality for EOTBL cancer by sex and UN region worldwide in 2022.

	Estimated new cancer cases and ASR per 100 000 people per year						Estimated cancer‐related mortality and ASR per 100 000 people per year					
UN region	Both sexes		Male		Female		Both sexes		Male		Female	
	Cases	ASR	Cases	ASR	Cases	ASR	Cases	ASR	Cases	ASR	Cases	ASR
Eastern Asia	71277	7.7	34308	7.2	36969	8.1	27234	2.9	17603	3.7	9631	2.1
Western Europe	4241	4.3	2279	4.6	1962	4	2384	2.4	1369	2.7	1015	2
Eastern Europe	6997	4.1	4756	5.6	2241	2.6	4545	2.6	3175	3.7	1370	1.6
Western Asia	5535	3.7	3867	4.7	1668	2.4	3128	2.1	2197	2.7	931	1.3
South‐Eastern Asia	13308	3.6	9132	5	4176	2.3	9313	2.5	6455	3.5	2858	1.5
Southern Europe	3072	3.3	1567	3.4	1505	3.3	1779	1.9	987	2	792	1.7
Australasia	429	2.7	194	2.4	235	3	230	1.4	118	1.5	112	1.4
Southern Africa	938	2.6	601	3.4	337	1.9	713	2.2	487	3	226	1.3
Northern America	4890	2.6	2245	2.4	2645	2.8	1644	0.88	828	0.88	816	0.87
Northern Europe	1455	2.6	741	2.7	714	2.6	476	0.86	274	0.99	202	0.73
Northern Africa	2984	2.5	2269	3.9	715	1.2	2483	2.1	1936	3.4	547	0.89
Pacific Islands	139	2.1	82	2.3	57	1.8	94	1.6	56	1.8	38	1.3
Caribbean	438	2	224	2	214	2	323	1.5	176	1.6	147	1.3
South America	4191	1.8	2143	1.9	2048	1.7	3025	1.3	1478	1.3	1547	1.3
South Central Asia	14383	1.4	8715	1.6	5668	1.1	12294	1.2	7489	1.5	4805	0.92
Eastern Africa	1475	0.84	771	0.89	704	0.79	1221	0.71	634	0.74	587	0.67
Central America	729	0.75	356	0.74	373	0.75	703	0.73	362	0.77	341	0.68
Middle Africa	372	0.56	255	0.79	117	0.34	297	0.46	206	0.64	91	0.27
Western Africa	852	0.54	445	0.57	407	0.51	760	0.49	405	0.52	355	0.45
World	137705	3.4	74950	3.7	62755	3.1	72646	1.8	46235	2.3	26411	1.3

*Note*: ASR, age‐standardized rate; UN, United Nations; EOTBL, early‐onset Tracheal, bronchial, and lung.

Regions such as North America displayed a pronounced disparity between mortality and incidence (ASMR: 0.88 vs. ASIR: 2.6 per 100 000 person‐years) (Figure 1C, Table S3), whereas Eastern Africa exhibited near equivalence (0.84 vs. 0.71). It is critical to interpret these ASIR‐ASMR differences strictly as ecological proxies. As demonstrated in epidemiological validation studies [20], such macro‐level gaps can roughly reflect survival disparities but cannot reliably infer stage distribution, treatment access, or definitive health system performance. Therefore, while wide gaps in Japan (0.56 vs. 3.39) and the USA (0.87 vs. 2.70) historically prompted hypotheses regarding healthcare infrastructure, they do not directly equate to system efficiency. Similarly, the minimal gaps observed in Afghanistan (1.62 vs. 1.51) and Morocco (4.45 vs. 4.43) highlight a severe population‐level mortality burden rather than specific clinical failures. These findings emphasize that ecological data must be supplemented with localized clinical evidence to accurately assess survival and access to care.

Pronounced sex‐based disparities were observed in the global burden of EOTBL cancer (Figure S1). Men bore a consistently higher burden, accounting for 74 950 new cases (ASIR, 3.7) and 46 235 deaths (ASMR, 2.3) globally. In contrast, women accounted for 62 755 new cases (ASIR, 3.1) and 26 411 deaths (ASMR, 1.3). Furthermore, this disparity exhibited substantial geographical variation. For instance, the ASIR in males markedly exceeded that in females across several regions, including North Africa, Western Asia, and Eastern Europe (Figure S1A). This disparity was most extreme in North Africa, where the male ASIR (3.9) was more than three times the female rate (1.2). A similar, although less pronounced, male‐predominant pattern was observed in Western Asia (male: 4.7; female: 2.4) and Eastern Europe (male: 5.6; female: 2.6). However, this pattern was reversed in other regions. In North America, the ASIR was higher in females (2.8) than in males (2.4). A similar female predominance was observed in Australia (female: 3.0 vs. male: 2.4). The Caribbean presents a unique exception, with no sex‐based disparity in incidence (2.0 for both sexes).

### 3.2 Disparities in EOTBL Burden According to the HDI

**FIGURE 2:**
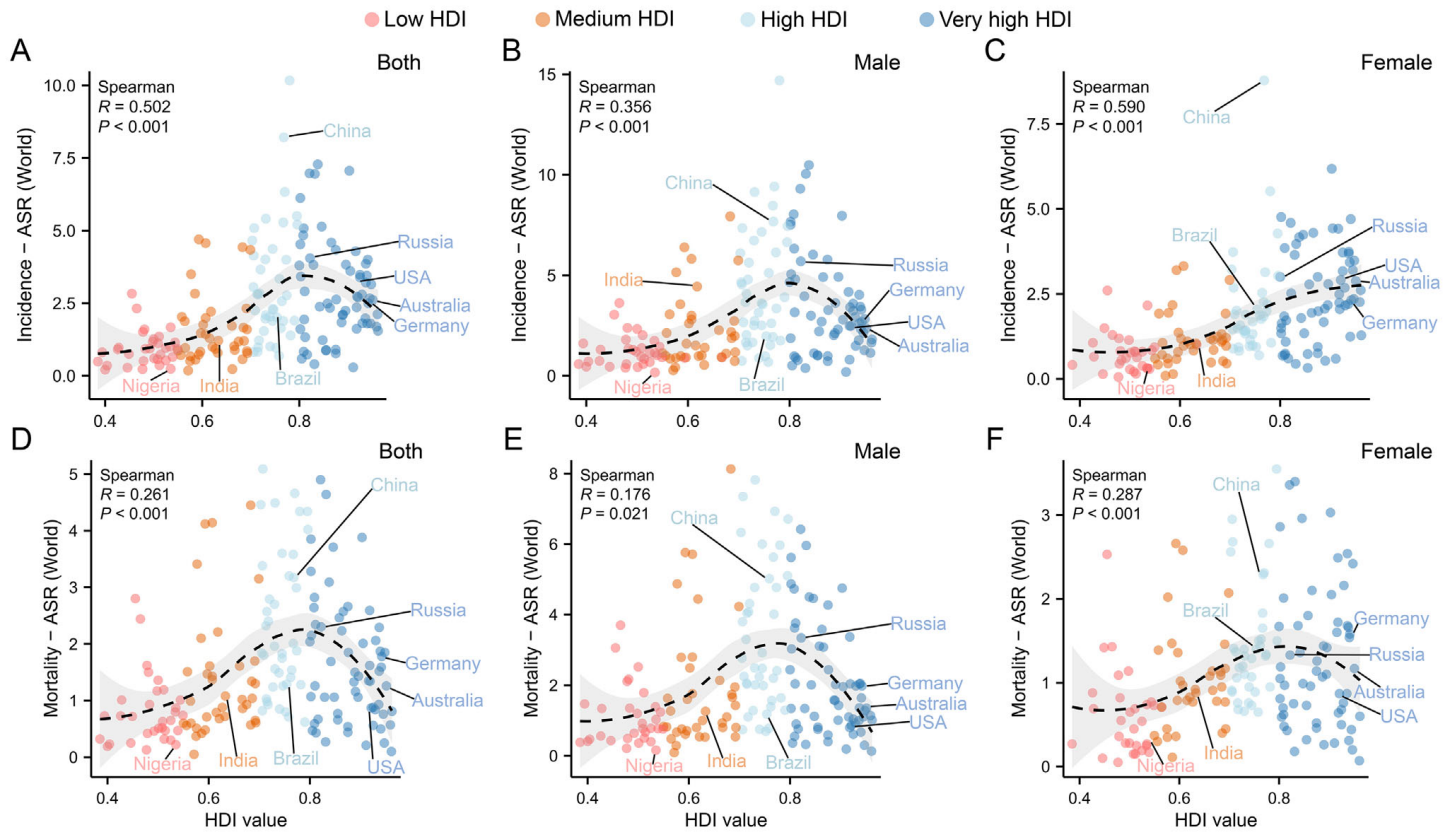
HDI‐related inequalities in the burden of EOTBL cancers, 2022. (A–C) Associations between the HDI and ASIR for both sexes (A), males (B), and females (C). (D–F) Associations between the HDI and ASMR for both sexes (D), males (E), and females (F). Each dot represents an individual country.

The global burden of EOTBL cancer exhibited a pronounced socioeconomic gradient that followed a distinct “inverted U‐shaped” pattern across the four tiers of the HDI in 2022 (Figure 2, Table S4). Notably, the disease burden was disproportionately concentrated in the High HDI tier, which accounted for approximately 62% of the global incident cases. Specifically, the High HDI tier recorded the highest incidence (85 548 new cases; ASIR 5.5 per 100 000 person‐years), followed by a decline in the Very High HDI tier (ASIR 3.4). In contrast, the Medium and Low HDI tiers exhibited substantially lower ASIRs of 1.6 and 0.88, respectively.

However, analyzing the MIR revealed a stark disparity in the population‐level mortality burden relative to incidence. While the High and Very High HDI groups maintained comparatively lower MIRs (approximately 0.47), the Low and Medium HDI tiers exhibited MIRs approaching 0.90 (0.88 and 0.89, respectively). While these ecological indices should not be directly equated with individual clinical prognoses or health‐system efficiency, this near‐unity ratio suggests that, despite lower absolute incidence rates, EOTBL cancer in less‐developed regions represents a disproportionately severe macroscopic burden. Such global disparities highlight complex, multifactorial heterogeneities in local socio‐environmental contexts that warrant further clinical investigation.

Furthermore, while a male predominance was evident across most tiers, the sex disparity narrowed significantly in the High HDI group (Figure 2B,E). The male‐to‐female incidence ratio was lowest in the High HDI tier (1.08), with wider gaps in the Medium (1.67) and Low (1.37) tiers. Contrary to the linear trend, both sexes followed an inverted U‐shaped trajectory, peaking at the High HDI level before declining in the Very High HDI group (Figure 2).

### 3.3 Temporal Trends of EOTBL Cancer in Selected Illustrative Countries

Given the substantial demographic influence on the disease burden, we analyzed the temporal trend of EOTBL in eight representative populous countries spanning six continents, covering all four HDI tiers. The selected countries included the USA, Germany, Australia, and Russia (Very High HDI); China and Brazil (High HDI); India (Medium HDI); and Nigeria (Low HDI) (Table 2). Although this primary panel captures the absolute disease burden driven by sheer demographic size, it may not fully encapsulate regions with the highest localized burden, diverse epidemiological patterns, or all distinct policy‐relevant contexts. Therefore, to ensure robust representation of diverse macroepidemiological contexts, we established an extended supplementary panel of eight additional nations (Japan, UK, Egypt, Indonesia, Bangladesh, Papua New Guinea, Ethiopia, and Pakistan). These countries were selected to represent unique epidemiological archetypes, extremely high specific burdens, and distinct sociocultural risk profiles that complement the primary population group. The detailed results for this supplementary cohort are presented in Figure S2 and Table S5.

**TABLE 2:** Changes in the incidence, mortality, and DALYs of EOTBL cancer across the eight main countries during 1990–2023 and 2019–2023.

Location	AAPC of incidence rate, % (95% CI)		AAPC of mortality rate, % (95% CI)		AAPC of DALYs rate, % (95% CI)	
	1990–2023	2019–2023	1990–2023	2019–2023	1990–2023	2019–2023
Both						
Australia	−1.68 (−1.84 to −1.53)	−3.27 (−3.67 to −2.91)	−2.37 (−2.54 to −2.22)	−3.49 (−3.95 to −3.11)	−2.33 (−2.49 to −2.18)	−3.41 (−3.83 to −3.06)
Brazil	−1.06 (−1.14 to −1.00)	0.53 (−0.11 to 1.16)	−1.16 (−1.23 to −1.10)	0.34 (−0.31 to 0.96)	−1.08 (−1.14 to −1.02)	0.61 (0.06–1.12)
China	−1.37 (−1.43 to −1.30)	1.86 (1.33 to 2.35)	−1.61 (−1.67 to −1.54)	1.49 (0.95–2.00)	−1.67 (−1.74 to −1.61)	1.57 (1.03–2.06)
Germany	−1.99 (−2.11 to −1.88)	−1.48 (−2.83 to −0.62)	−2.64 (−2.74 to −2.55)	−2.62 (−2.89 to −2.26)	−2.52 (−2.65 to −2.41)	−1.19 (−2.50 to −0.31)
India	1.18 (0.95–1.40)	2.00 (1.47–3.25)	1.13 (0.9–1.35)	1.92 (1.4–3.07)	1.12 (0.89–1.35)	1.88 (1.36–3.18)
Nigeria	−0.07 (−0.19 to 0.04)	5.82 (4.84–6.73)	−0.12 (−0.27 to 0.03)	5.47 (4.18–6.69)	−0.11 (−0.25 to 0.02)	5.62 (4.48–6.67)
Russia	−2.96 (−3.09 to −2.81)	−4.09 (−4.70 to −3.53)	−3.14 (−3.26 to −2.98)	−4.35 (−4.96 to −3.86)	−3.11 (−3.24 to −2.95)	−4.35 (−4.99 to −3.83)
USA	−3.67 (−3.76 to −3.60)	−2.34 (−3.25 to −1.09)	−3.81 (−3.91 to −3.73)	−2.52 (−3.50 to −1.13)	−3.76 (−3.86 to −3.67)	−2.47 (−3.47 to −1.11)
Male						
Australia	−2.08 (−2.36 to −1.84)	−3.28 (−5.87 to −1.37)	−2.79 (−3.00 to −2.60)	−3.21 (−3.96 to −2.77)	−2.74 (−2.95 to −2.56)	−3.20 (−3.89 to −2.76)
Brazil	−1.78 (−1.89 to −1.70)	0.73 (−0.19 to 1.46)	−1.87 (−1.97 to −1.79)	0.59 (−0.24 to 1.28)	−1.81 (−1.90 to −1.73)	0.63 (−0.16 to 1.29)
China	−1.08 (−1.16 to −1.02)	1.73 (1.11–2.23)	−1.23 (−1.30 to −1.17)	1.50 (0.89–2.00)	−1.31 (−1.40 to −1.23)	1.38 (0.61–2.00)
Germany	−2.92 (−3.11 to −2.74)	−1.74 (−3.13 to 0.42)	−3.40 (−3.52 to −3.29)	−2.48 (−2.83 to −2.03)	−3.30 (−3.46 to −3.12)	−2.46 (−3.03 to −1.05)
India	1.51 (1.06–1.89)	4.85 (1.29–8.07)	1.45 (0.92–1.84)	4.64 (1.12–7.93)	1.46 (0.99–1.84)	4.60 (1.21–7.75)
Nigeria	−0.22 (−0.38 to −0.07)	4.63 (3.35–5.92)	−0.24 (−0.40 to −0.10)	4.52 (3.25–5.82)	−0.24 (−0.38 to −0.12)	4.46 (3.38–5.54)
Russia	−3.42 (−3.63 to −3.13)	−3.95 (−4.16 to −3.79)	−3.51 (−3.72 to −3.20)	−4.09 (−4.29 to −3.93)	−3.44 (−3.66 to −3.08)	−4.05 (−4.26 to −3.90)
USA	−4.11 (−4.23 to −3.98)	−1.91 (−3.22 to −0.11)	−4.19 (−4.31 to −4.06)	−2.00 (−3.24 to −0.24)	−4.13 (−4.27 to −4.00)	−1.80 (−3.18 to 0.10)
Female						
Australia	−1.02 (−1.19 to −0.82)	−1.93 (−3.31 to −0.38)	−1.75 (−1.93 to −1.55)	−2.25 (−3.68 to −0.78)	−1.68 (−1.86 to −1.49)	−2.13 (−3.48 to −0.73)
Brazil	0.07 (0.01–0.14)	0.51 (−0.01 to 0.97)	−0.04 (−0.11 to 0.03)	0.17 (−0.40 to 0.67)	−0.02 (−0.08 to 0.05)	0.45 (−0.10 to 0.93)
China	−1.74 (−1.79 to −1.68)	2.43 (1.92–2.86)	−2.14 (−2.20 to −2.09)	1.90 (1.45–2.31)	−2.22 (−2.28 to −2.17)	1.90 (1.42–2.31)
Germany	−0.14 (−0.27 to −0.01)	−2.58 (−3.07 to −1.43)	−0.89 (−0.99 to −0.77)	−2.50 (−2.91 to −1.39)	−0.82 (−1.00 to −0.68)	−2.45 (−3.24 to −1.45)
India	1.55 (1.44–1.64)	4.95 (3.96–5.80)	1.47 (1.37–1.56)	4.75 (3.91–5.54)	1.41 (1.31–1.50)	4.64 (3.84–5.37)
Nigeria	0.57 (0.47–0.67)	7.29 (6.40–8.06)	0.55 (0.44–0.65)	7.05 (6.15–7.89)	0.48 (0.38–0.59)	7.00 (6.07–7.84)
Russia	−0.89 (−1.02 to −0.75)	−4.30 (−5.32 to −3.49)	−1.17 (−1.34 to −1.00)	−4.59 (−5.72 to −3.56)	−1.20 (−1.35 to −1.05)	−4.51 (−5.58 to −3.61)
USA	−3.11 (−3.20 to −3.03)	−2.21 (−3.31 to −1.53)	−3.27 (−3.39 to −3.19)	−2.51 (−3.85 to −1.72)	−3.23 (−3.33 to −3.15)	−2.40 (−3.67 to −1.65)

*Note*: DALYs, disability‐adjusted life years; EOTBL, early‐onset Tracheal, bronchial, and lung; AAPC, average annual percent change; CI, confidence interval.

**FIGURE 3:**
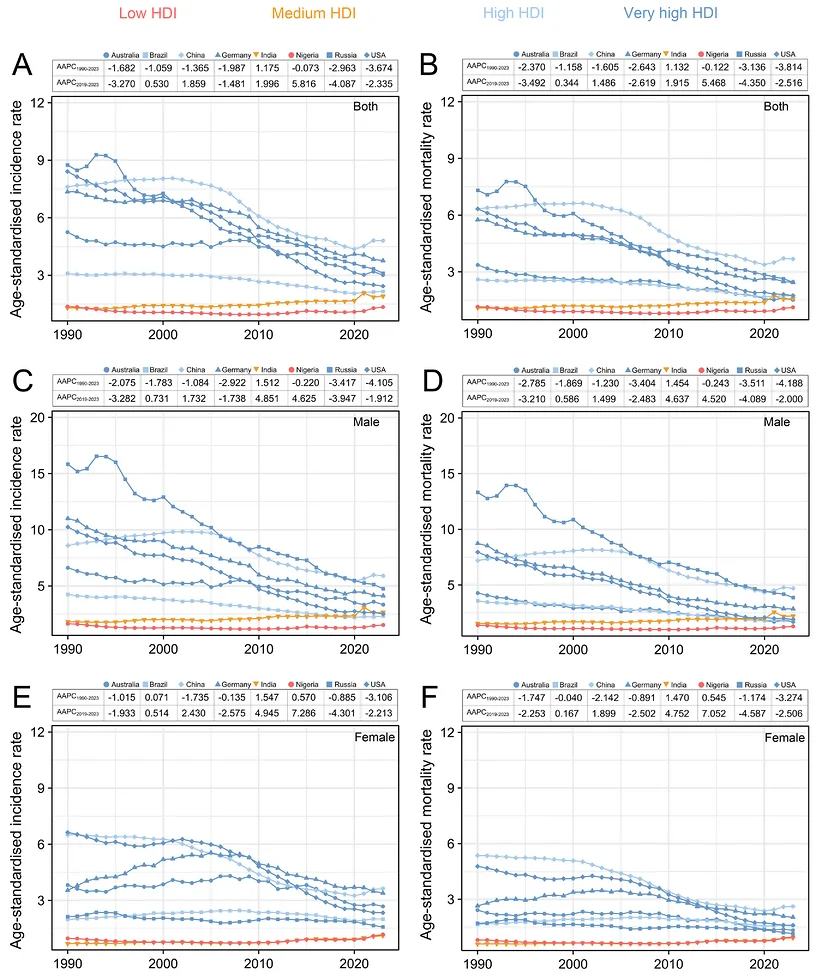
Temporal trends in the burden of EOTBL cancers from 1990 to 2023. (A, C, E) Trends in the ASIR for both sexes (A), males (C), and females (E). (B, D, F) Trends in the ASMR for both sexes (B), males (D), and females (F).

Focusing on the primary cohort, the temporal trend of EOTBL cancer incidence from 1990 to 2023 showed significant variations across the eight main countries (Figure 3; Tables S7, S9, and S11). In 1990, the highest ASIRs were recorded in Russia (8.75 per 100 000 person‐years), the USA (8.41), and China (7.61). By 2023, this ranking had shifted, with China (4.80), Germany (3.76), and Russia (3.10) representing the highest incidence burdens (Table S6). The ASIR trajectories also diverged over time. The USA, Germany, Australia, and Russia (with Very High HDI) exhibited sustained declines throughout the study period. China (annual percentage change [APC]: 3.45 [95% CI 2.69–4.15]) and Brazil (APC: 1.70 [95% CI 0.49–3.61]) (High HDI) showed an early downward trend that reversed after an inflection point around 2020, leading to rising incidence thereafter (Table S6). In contrast, India (AAPC: 1.18 [95% CI 0.95–1.40]) (Medium HDI) experienced an overall upward trend in incidence rates from 1990 to 2023.

Sex‐specific ASIR trajectories in these eight countries also differed markedly. In males, patterns largely paralleled the overall patterns, with Russia maintaining notably higher rates than other countries from 1990 to 2005 (Figure 3C). In contrast, female incidence followed divergent trajectories (Figure 3E). In Russia, female rates remained consistently low and stable throughout the study period (AAPC: −0.89 [95% CI −1.02 to −0.75]) (Table 2). Germany, Australia, and Brazil showed a parabolic‐like trajectory, with rates rising from 1990 to a peak around 2007, followed by a decline until 2023. Meanwhile, the USA and China showed initially overlapping female incidence trends that diverged after 2013; the USA rates continued to decline, whereas Chinese rates began to rise again after 2020 (APC: 3.95 [95% CI 3.23–4.57]). By comparison, India (APC: 1.55 [95% CI 1.44–1.64]) and Nigeria (APC: 0.57 [95% CI 0.47–0.67]) demonstrated nearly identical and steadily increasing female incidence trends over the entire period (Table S8).

Temporal ASMR trends were similarly analyzed across the eight countries (Figure 3; Tables S8, S10, and S12). In 1990, the highest ASMRs were observed in Russia (7.31 per 100 000 person‑years), USA (6.33), and China (6.33). By 2023, this ranking had shifted, with China (3.68), Russia (2.45), and Germany (2.44) displaying the highest mortality burdens (Table S5). Mortality trends (both overall and stratified by sex) closely mirrored the corresponding incidence patterns observed (Figure 3). This alignment indicates that changes in mortality burden largely corresponded to shifts in disease incidence, reinforcing the close epidemiological linkage between diagnosis and outcome across these populations. In addition, to reduce heterogeneity within the early‐onset population, we stratified individuals aged 15–49 years into narrower age groups and compared age‐specific incidence and mortality in 1990 and 2023 across the eight primary representative countries. These results are presented in Figures S3 and S4.

### 3.4 Temporal Trajectories in the Contribution of Major Risk Factors for EOTBL Across Selected Illustrative Countries

Based on the observed epidemiological patterns, we initially examined 16 risk factors contributing to the burden of EOTBL cancer across the 16 countries (Table 3, Figures S5 and S6, and Table S13). This broad analysis identified four primary risk factors (smoking, secondhand smoke, ambient particulate matter pollution, and household air pollution from solid fuels) as the dominant contributors. Subsequently, we analyzed the changes in these four primary risk factors between sexes from 1990 to 2023 across the primary panel of eight countries.

**TABLE 3:** Percentage contribution of the four primary risk factors to DALYs of EOTBL cancer by sex across the eight main countries in 2023.

Location	Risk factors			
	Ambient particulate matter pollution,% (95% UI)	Household air pollution from solid fuels, % (95% UI)	Secondhand smoke, % (95% UI)	Smoking, % (95% UI)
Both				
Australia	3.0 (1.4–5.0)	0.0 (0.0–0.4)	8.0 (6.3–9.6)	50.1 (41.2–60.1)
Brazil	8.8 (5.5–12.5)	0.4 (0.0–1.4)	7.8 (6.1–9.6)	28.0 (24.5–32.1)
China	20.5 (14.0–27.5)	2.3 (0.0–7.6)	11.8 (9.4–14.5)	48.1 (42.1–54.1)
Germany	4.0 (1.9–6.5)	0.1 (0.0–0.6)	9.6 (7.6–11.7)	59.0 (52.9–63.9)
India	16.8 (11.1–23.0)	12.8 (7.9–18.4)	8.6 (6.7–10.6)	20.9 (15.4–26.1)
Nigeria	15.7 (10.5–21.2)	15.9 (10.3–22.6)	3.6 (2.8–4.5)	8.8 (4.8–14.0)
Russia	6.5 (3.8–9.6)	0.1 (0.0–0.4)	8.6 (6.9–10.5)	65.6 (60.1–69.7)
USA	3.1 (1.4–5.2)	0.0 (0–0.2)	7.8 (6.2–9.3)	56.0 (47.0–64.6)
Male				
Australia	3.1 (1.4–5.1)	0.0 (0.0–0.3)	8.8 (6.9–10.4)	52.9 (45.7–60.0)
Brazil	8.7 (5.5–12.4)	0.3 (0.0–1.2)	8.9 (6.7–10.9)	32.2 (26.0–38.5)
China	20.5 (14.0–27.4)	1.9 (0–6.9)	8.0 (6.1–10.1)	69.9 (64.8–73.7)
Germany	4.0 (1.9–6.5)	0.1 (0.0–0.6)	10.2 (8.1–12.4)	65.7 (59.1–72.8)
India	17.5 (11.5–23.8)	11.7 (7.1–17.4)	7.9 (6.2–9.7)	28.4 (21.9–34.6)
Nigeria	17.5 (11.8–23.7)	14.6 (8.9–21.3)	3.4 (2.6–4.3)	16.1 (10.2–23.1)
Russia	6.5 (3.7–9.5)	0.1 (0.0–0.3)	7.3 (5.7–9.1)	74.8 (69.7–78.6)
USA	3.1 (1.4–5.2)	0.0 (0.0–0.1)	8.7 (7.0–10.4)	59.6 (51.2–66.1)
Female				
Australia	3.0 (1.4–5.0)	0.1 (0.0–0.5)	7.0 (5.4–8.7)	46.8 (34.0–60.8)
Brazil	8.8 (5.5–12.5)	0.6 (0.0–1.7)	6.7 (5.1–8.3)	23.3 (17.0–30.0)
China	20.4 (13.6–27.6)	3.1 (0.1–9.4)	19.1 (15.9–21.9)	7.1 (4.2–11.1)
Germany	4.0 (1.9–6.5)	0.1 (0.0–0.9)	8.7 (6.9–10.6)	49.4 (34.6–61.6)
India	15.1 (10.0–21.0)	15.6 (9.9–22.0)	10.3 (8.3–12.5)	2.2 (1.4–3.4)
Nigeria	14.0 (9.3–19.2)	17.3 (11.3–24.0)	3.8 (2.9–4.7)	1.3 (0.5–2.6)
Russia	6.6 (3.9–9.8)	0.1 (0.0–0.5)	12.7 (10.2–15.1)	34.5 (29.0–44.3)
USA	3.1 (1.4–5.2)	0.0 (0.0–0.2)	6.7 (5.4–8.1)	51.7 (39.8–62.6)

*Note*: DALYs, disability‐adjusted life years; EOTBL, early‐onset Tracheal, bronchial, and lung; UI, uncertainty interval.

**FIGURE 4:**
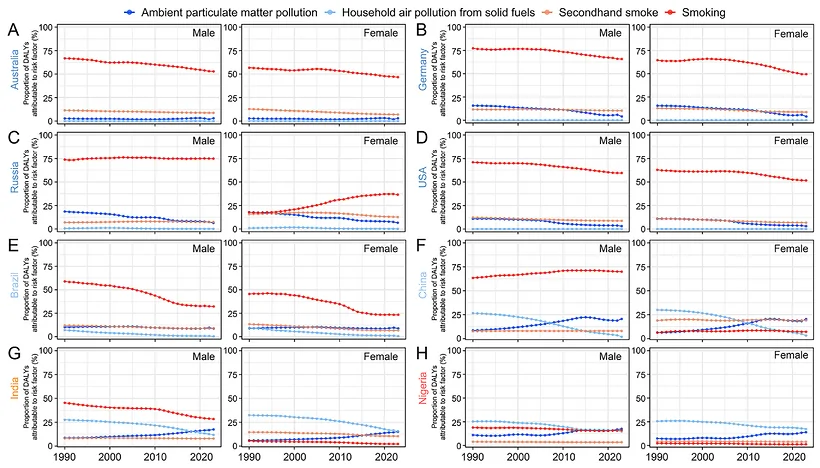
Proportion of EOTBL cancer DALYs attributable to four primary risk factors across eight major populous countries from 1990 to 2023. The evaluated risk factors include ambient particulate matter pollution, household air pollution from solid fuels, secondhand smoke, and active smoking. Data are presented for (A) Australia, (B) Germany, (C) Russia, (D) USA, (E) Brazil, (F) China, (G) India, and (H) Nigeria.

As presented in Figure 4, their relative contributions exhibited notable heterogeneity across this primary cohort, resulting in distinct patterns of attributable burdens. In both males and females across the USA, Germany, Australia, Brazil, and Russia, smoking remained the predominant contributor to the EOTBL cancer burden (20%–76%). The contributions of the other three risk factors were notably smaller (1%–12%). As the leading factor, smoking exhibited a gradually declining trend in its contribution to disease burden among both sexes in the USA, Germany, and Australia (Figure 4A,B,D). In contrast, smoking maintained a persistently high contribution among Russian males throughout the period, while its impact among Russian females increased sharply, rising from 15% in 2000% to 30% by 2023 (Figure 4C). Among males in China and India, smoking remained the predominant risk factor for EOTBL cancer. The second leading contributor shifted from household air pollution from solid fuels in 1990 to ambient particulate matter pollution by 2023 (Figure 4F,G). Among females in the two countries, ambient particulate matter pollution similarly replaced household air pollution from solid fuels as the primary contributor over the same period. The secondary risk factor, however, differed between the two nations, with secondhand smoke ranking second in China, and household air pollution from solid fuels remaining the second leading factor in India. In Nigeria, as in India, the trajectories of household air pollution from solid fuels and ambient particulate matter pollution had similar contributions to disease burden and closely paralleled each other for both sexes. However, a notable distinction was that smoking consistently remained the predominant risk factor among Nigerian males over the entire study period (Figure 4H). Furthermore, the temporal trends of these four primary risk factors across the supplementary panel of the remaining eight countries are detailed in Figure S7. Collectively, these results highlight the significant heterogeneity in the major risk factors for EOTBL cancer across different countries, reflecting variations in environmental, behavioral, and transitional epidemiologic contexts.

## 4 Discussion

This study systematically assessed the global disease burden of EOTBL cancer in individuals aged 15–49 years. By integrating contemporary data from GLOBOCAN 2022 and longitudinal trends from a primary cohort of representative populous countries alongside an extended supplementary panel from the GBD 2023 study, we present up‐to‐date evidence on the magnitude, geographical distribution, and key determinants of this growing public health challenge. Our analysis highlights a landscape characterized by profound disparities, with Eastern Asia and China emerging as regions of increasingly severe disease burden. Furthermore, these inequalities manifest across multiple dimensions, including pronounced differences among geographical regions, persistent disparities between sexes and human development levels, and distinct variations over time. It should also be noted that the underlying risk factor profiles have undergone distinct shifts across these diverse national settings. These evolving epidemiological patterns collectively underscore the critical need for stratified, context‐specific prevention and control strategies.

Similar to most malignancies, the incidence of TBL cancer rises substantially with age, with a median reported age at diagnosis of approximately 71 years [21]. Although EOTBL represents a minor subset of total lung cancer cases, it presents with distinct clinicopathological and prognostic features [9]. These distinguishing characteristics reinforce its recognition as a unique clinical entity requiring further investigation. Epidemiological evidence indicates that non‐smokers, females, and adenocarcinoma histology predominate in early‐onset lung cancers [22, 23]. In the GLOBOCAN 2022‐based analysis, Eastern Asia reported approximately 71 277 new cases of EOTBL cancer, accounting for 53.8% of global incidence. This notable proportion can be attributed not only to the region's massive population base but also to the expanding use of advanced diagnostic modalities, particularly the increasing adoption of low‐dose computed tomography (LDCT) in screening programs and routine clinical practice [24, 25, 26]. In addition to Eastern Asia, Eastern Europe, where detailed epidemiological studies on EOTBL cancer remain scarce, also demonstrated a high ASIR (4.1) and severe ASMR (2.6) for this cancer (Table 1), indicating a similarly substantial disease burden in the region. The disparity between incidence and mortality rates serves as an ecological proxy. While previous studies have suggested statistical associations between such temporal trends and broad factors, such as cancer spectrum variations or regional socioeconomic status [27], these macro‐level indicators cannot robustly infer specific health‐system performance features, such as stage distribution or treatment access. Regions such as North America and Japan display pronounced disparities (lower ASMR relative to ASIR), which aligns with patterns typically observed in highly developed settings [28]. In contrast, the near equivalence of ASIR and ASMR in regions such as Eastern Africa and countries including Afghanistan and Morocco highlights deep geographical disparities in disease outcomes. This profound global divergence underscores the urgent need for granular clinical‐level data to better understand the distinct diagnostic and therapeutic challenges across different regions.

Our analysis, stratified by HDI, reveals a pronounced socioeconomic gradient in the burden of EOTBL cancer, consistent with patterns observed during the epidemiological transition [29, 30]. The highest disease burdens were observed in the High and Very High HDI regions, likely attributable to more advanced detection systems and earlier exposure to lifestyle and environmental risk factors associated with economic development [31]. Based on these observations, we focused our longitudinal analysis on a primary cohort of eight major populous countries spanning all four HDI tiers, complemented by a supplementary panel of eight representative nations to capture localized high‐burden settings and distinct policy‐relevant contexts. Using a data‐driven approach, we identified four primary risk factors (active smoking, secondhand smoke, ambient particulate matter, and household air pollution) that cumulatively accounted for the vast majority (> 85%–90%) of the risk‐attributable EOTBL burden. Other factors, such as occupational exposure, contributed marginally at the macro‐national level (often < 1%–5%), largely because their long latency periods primarily affect older rather than early‐onset populations.

Countries with Very High HDI levels, including the USA, Germany, and Australia, exhibited sustained declines in the overall and male‐specific disease burden of EOTBL throughout the 1990–2023 period. We also observed distinct patterns in the contributions of different risk factors across specific countries. In these nations, where smoking has maintained a predominant role in the attributable burden, the concurrent decline in smoking‐attributable burden may be consistent with the potential contribution of historical tobacco‐control efforts, although causal attribution cannot be established in the present ecological analysis [32]. However, a striking exception within the Very High‐HDI tier was Russia. Despite the global trend toward effective tobacco control, the attributable burden of smoking has remained persistently high among Russian men and has increased significantly among Russian women in recent decades. This anomaly highlights the urgent need for sex‐specific tobacco interventions in this region.

It is also noteworthy that although China and Brazil are both classified as High‐HDI countries, they exhibit markedly different EOTBL cancer burdens, with China showing substantially higher incidence and mortality rates, indicating that HDI alone cannot fully explain country‐level heterogeneity in EOTBL epidemiology. China's elevated burden may reflect its historically large smoking population, high smoking prevalence among men, substantial ambient particulate matter pollution during rapid industrialization, and a tobacco control trajectory contrasting with Brazil's successful FCTC (World Health Organization Framework Convention on Tobacco Control)‐aligned anti‐tobacco campaigns, including advertising restrictions, pictorial health warnings, and tobacco taxation, which have reduced smoking prevalence and may partly explain Brazil's lower burden [33, 34]. Despite these baseline differences, both countries exhibited a renewed upward trend in EOTBL burden from 2019 to 2023 after a prolonged decline. In China, this increase is likely related to population‐wide LDCT screening and widespread chest CT use during the COVID‐19 pandemic, which enhanced early case ascertainment [35, 36], whereas Brazil's recent upward shift remains unclear. Risk‐factor profiles also differed: smoking dominated in Brazil (Figure 4E), while in China (Figure 4F), smoking led among males, and ambient PM2.5 exposure and secondhand smoke were major contributors among females. These findings suggest distinct EOTBL trajectories within the same HDI category due to differences in exposures, screening, and cancer control policies.

Furthermore, our temporal analysis revealed a classic epidemiological indicator of industrial and energy transitions in developing nations, such as China: the crossover effect between environmental risk factors [37]. Over the past three decades, ambient particulate matter pollution has progressively surpassed household air pollution from solid fuels as a leading driver in females. This shift may reflect broader socioeconomic, industrial, environmental, and household energy transitions, including the gradual replacement of indoor solid fuels with cleaner domestic energy sources, while populations remain exposed to substantial ambient particulate matter pollution [38]. Additionally, in Medium‐ and Low‐HDI countries such as India and Nigeria, an alarming pattern has emerged: a disproportionately high mortality burden relative to incidence. This indicates a dual challenge, where these nations face a rising incidence of EOTBL while their health systems remain under‐resourced and struggle to provide timely care [39].

The distinct clinical and molecular characteristics of early‐onset lung cancer necessitate a dedicated approach to its management. Our recent findings suggest that the etiology of these malignancies differs from that of later‐onset diseases, with a more prominent role for specific somatic driver alterations (e.g., *ALK* fusions and *ERBB2* mutations) and hereditary predisposition [7, 40, 41]. This biological distinctiveness, coupled with the diagnosis occurring during an individual's peak productive years, amplifies the personal, familial, and socioeconomic consequences. Consequently, current screening paradigms warrant re‐evaluation. Although overdiagnosis must be mitigated, early screening of targeted high‐risk subgroups, especially those with a strong family history, genetic susceptibility, or massive environmental exposure, could facilitate timely detection and improve outcomes [42, 43, 44]. The existing disparities in international screening guidelines highlight the pressing need for evidence specifically derived from younger populations.

This study has certain limitations. First, although GLOBOCAN and GBD share similar foundational registry data, they operate on distinct modeling frameworks. Consequently, our analyses using GLOBOCAN 2022 (cross‐sectional precision) and GBD 2023 (longitudinal continuity) were conducted in strictly separate, parallel tracks. By explicitly avoiding quantitative cross‐pooling and direct comparison of their outputs, we respected their architectural constraints, meaning that the findings should be interpreted exclusively within their respective analytical contexts [45]. Second, the accuracy of global estimates inherently depends on the quality of the underlying cancer registry. Underdiagnosis and underreporting in regions with less developed health infrastructure likely lead to an underestimation of the true burden, particularly in some low‐ and middle‐income countries [46]. Finally, our analyses were based on population and macroepidemiological attributes. We lacked granular, individual‐level data on specific screening exposures, clinical stages, and individual causality, meaning that the inferred impacts of widespread LDCT utilization or COVID‐19 disruptions remain interpretive based on current ecological trends.

## 5 Conclusions

This study provides an updated and comprehensive assessment of the global burden and epidemiological trends of EOTBL cancer and reveals significant disparities based on geography, human development, and sex. Although High‐HDI regions have the highest incidence rates, Low‐ and Medium‐HDI regions bear a disproportionately high mortality burden, largely attributable to later‐stage diagnosis and constrained access to timely and effective treatment. Although the incidence is generally higher in males, this historical pattern is reversing in some High‐HDI regions, signaling an important epidemiological shift. Furthermore, the leading risk factors exhibit considerable geographic variations, with active smoking remaining the predominant driver in many populations, whereas air pollution (both ambient and household) constitutes a major and comparable contributor in other populations. Consequently, addressing the EOTBL cancer burden requires tailored, multipronged public health strategies. These must integrate evidence‐based primary prevention, enhanced early detection programs, and equitable access to diagnostic and therapeutic services, all of which must be carefully calibrated to address distinct regional risk profiles and specific demographic disparities.

## Author Contributions

Y.T. and X.Y. were involved in the conceptualization and design of the study. X.Y. was the guarantor. Y.T., W.Z., and Y.L. were involved in data acquisition. W.Z. and Y.T. performed the statistical analyses and designed the figures. Y.T. and X.Y. provided funding. Y.T., W.Z., and Y.L. wrote the first draft of the manuscript. All authors revised the manuscript for important intellectual content and approved the final version.

## Funding

This study was supported by a grant from the Natural Science Foundation of Liaoning Province (#2025‐MS‐213) to Y.T. and the National Natural Science Foundation of China (#82573802) to X.Y.

## Conflicts of Interest

The authors declare no conflicts of interest.

## Supporting information




**Supporting File**: advs77778‐sup‐0001‐SuppMat.docx.

## Data Availability

The data that support the findings of this study are available from the corresponding author upon reasonable request.
