# Supplementary material for: Global Burden and Temporal Trends of Early‐Onset Tracheal, Bronchial, and Lung Cancer: An Analysis of GLOBOCAN 2022 and GBD 2023 Data
Source: Adv Sci (Weinh). 2026 Sep 16:e77778. Online ahead of print. doi: 10.1002/advs.77778 (PMC13583219; doi:10.1002/advs.77778)
Supplement: Supplementary file 1 [file ADVS-9999-e77778-s001.docx]

**SUPPLEMENTARY INFORMATION**

**Global Burden and Temporal Trends of Early-Onset Tracheal, Bronchial, and Lung Cancer: An Analysis of GLOBOCAN 2022 and GBD 2023 Data**

Ye Tian, Weihao Zhang, Yao Lu, Bruce F. O'Hara, Jingjing Hu, Hanqi Feng, Jing Di, Lei Zhang, and Xueying Yang.

Supplementary Information associated with this article include:

Figures S1-S7

Tables S1-S13

**Supplementary Figures**

**Figure S1. Sex disparities in ASIR (A) and ASMR (B) of EOTBL cancers across regions in 2022.** (ASIR: age-standardized incidence rate; ASMR: age-standardized mortality rate; EOTBL: Early-onset tracheal, bronchial, and lung.)

**Figure S2. Temporal trends in the burden of EOTBL cancers across a supplementary panel of eight additional representative countries from 1990 to 2023.** (**A, C, E**) Trends in the ASIR for both sexes (**A**), males (**C**), and females (**E**). (**B, D, F**) Trends in the ASMR for both sexes (**B**), males (**D**), and females (**F**).

**Figure S3. Age-specific incidence rates of EOTBL cancers across 5-year age groups (15–19, 20–24, 25–29, 30–34, 35–39, 40–44, and 45–49 years) in 1990 and 2023.** (**A-H**) Data are presented for (**A**) Australia, (**B**) Germany, (**C**) Russia, (**D**) USA, (**E**) Brazil, (**F**) China, (**G**) India, and (**H**) Nigeria.

**Figure S4. Age-specific mortality rates of EOTBL cancers across 5-year age groups (15–19, 20–24, 25–29, 30–34, 35–39, 40–44, and 45–49 years) in 1990 and 2023.** (**A-H**) Data are presented for (**A**) Australia, (**B**) Germany, (**C**) Russia, (**D**) USA, (**E**) Brazil, (**F**) China, (**G**) India, and (**H**) Nigeria.

**Figure S5.** **Percentage contribution of various risk factors to EOTBL cancer DALYs across eight major populous countries in 2023.** Data are presented separately for (**A**) female and (**B**) male. (DALYs: disability-adjusted life-years.)

**Figure S6.** **Percentage contribution of various risk factors to EOTBL cancer DALYs across a supplementary panel of eight additional representative countries in 2023.** Data are presented separately for (**A**) female and (**B**) male. (DALYs: disability-adjusted life-years.)

**Figure S7.** **Proportion of EOTBL cancer DALYs attributable to four primary risk factors across additional eight representative countries from 1990 to 2023.** The evaluated risk factors include ambient particulate matter pollution, household air pollution from solid fuels, secondhand smoke, and smoking. Data are presented for (**A**) Japan, (**B)** UK, (**C)** Egypt, (**D**) Indonesia, (**E**) Bangladesh, (**F)** Papua New Guinea, (**G**) Ethiopia, and (**H**) Pakistan.

**Supplementary Tables**

**Table S1. Criteria-based selection framework for the 16-country analytical panel**

| **Country** | **Panel** | **Continent/**  **region represented** | **HDI tier** | **Selection criterion met** | **Selection rationale** |
| --- | --- | --- | --- | --- | --- |
| Australia | Primary panel | Australasia | Very High HDI | Geographic representation; HDl representation; policy diversity; distinct exposure profile | Selected to represent Oceania and a very high HDI setting with strong cancer-control policies and distinctive risk-factor profiles. |
| Brazil | Primary panel | South America | High HDI | Geographic representation; population size; HDI representation; health-system heterogeneity | Selected as the most populous country in South America, representing a high HDI Latin American setting with heterogeneous regional health-system capacity. |
| China | Primary panel | Eastern Asia | High HDI | Population size; geographic representation; high EOTBL burden; health-system transition | Selected as a populous country with a major contribution to the global EOTBL burden and rapid socioeconomic, epidemiological, and health-system transition |
| Germany | Primary panel | Western Europe | Very High HDI | HDI representation; geographic representation; cancer surveillance capacity; policy diversity | Selected to represent a very high HDI European country with mature health system infrastructure, established cancer surveillance capacity, and a distinct cancer-control context |
| India | Primary panel | South Central Asia | Medium HDI | Population size; HDI representation; high absolute burden; geographic representation | Selected as one of the most populous countries worldwide, representing a medium HDI setting with substantial demographic weight and a large absolute EOTBL burden |
| Nigeria | Primary panel | Western Africa | Low HDI | Population size; low HDI representation; geographic representation; health-system heterogeneity | Selected as the most populous country in Africa and as a low HDI setting, representing rapidly growing demographic pressure and limited cancer control resources |
| Russia | Primary panel | Eastern Europe | Very High HDI | Population size; HDI representation; distinct exposure profile; high tobacco-related burden | Selected as a populous very high HDI country with a historically high tobacco-related disease burden and distinct temporal patterns in cancer risk factors. |
| USA | Primary panel | Northern America | Very High HDI | Geographic representation; HDI representation; Policy diversity; Cancer-control infrastructure | Selected to represent a very high HDI country with advanced cancer-control infrastructure, large population size, and distinct tobacco-control and screening contexts |
| Bangladesh | Supplementary panel | South Central Asia | Medium HDI | Regional complementarity; population size; high absolute burden; distinct exposure profile | Selected to complement India by representing a medium HDI South Asian setting with large population, substantial EOTBL burden, and distinct tobacco exposure and health‑system characteristics. |
| Egypt | Supplementary panel | Northern Africa | High HDI | Regional complementarity; HDI representation; high absolute burden; geographic representation | Selected to represent North Africa and a high-HDI setting with region-specific EOTBL burden patterns and distinct sociocultural and exposure profiles. |
| Ethiopia | Supplementary panel | Eastern Africa | Low HDI | Regional complementarity; low HDI representation; population size; health‑system heterogeneity | Selected to complement Nigeria by representing a low HDI East African setting with large population, limited cancer‑control resources, and growing demographic pressure, enhancing regional diversity within Africa. |
| Indonesia | Supplementary panel | South-Eastern Asia | High HDI | Regional complementarity; population size; high EOTBL burden; distinct exposure profile | Selected because of its large population, substantial regional EOTBL burden, and policy-relevant tobacco exposure profile in Southeast Asia. |
| Japan | Supplementary panel | Eastern Asia | Very High HDI | Regional complementarity; ageing population; high absolute burden; distinct temporal trend | Selected to complement the primary panel by representing an ageing, very high HDI East Asian population with high absolute EOTBL burden and distinct temporal patterns |
| Pakistan | Supplementary panel | South Central Asia | Low HDI | Regional complementarity; population size; low HDl representation; high absolute burden | Selected to complement India by representing a low HDI South Asian setting with large population size and substantial EOTBL burden |
| Papua New Guinea | Supplementary panel | Pacific Islands | Medium HDI | Regional complementarity; geographic representation; distinct exposure profile; health‑system heterogeneity | Selected to complement Australia by representing a medium HDI Pacific Island setting with distinct geographic and epidemiological characteristics, capturing under‑represented island populations and diverse health‑system capacity. |
| UK | Supplementary panel | Northern Europe | Very High HDI | Regional complementarity; policy diversity cancer registration capacity; distinct temporal trend | Selected to complement Germany by representing a very high HDI setting with long-standing tobacco-control policies, established cancer registration systems, and distinct temporal trend patterns. |

Note: Counties were selected using a prespecified, transparent criteria-based framework. The primary panel consisted of eight populous countries selected to maximize geographic diversity HDI representation, demographic weight, and health-system heterogeneity. The supplementary panel included eight additional countries selected to capture region-specific E0TBL burden, divergent temporal tends, exposure-profile diversity, and policy heterogeneity across HDI strata. HDI categories were based on the United Nations Human Development Index classification used in this study.

**Table S2. Classification of 185 countries and territories into four HDI categories based on their 2023 HDI values.**

| **HDI category** | **Country** |
| --- | --- |
| Very High HDI | Argentina, Australia, Austria, Bahamas, Bahrain, Belgium, Brunei Darussalam, Belarus, Canada, Chile, Costa Rica, Croatia, Cyprus, Czechia, Denmark, Estonia,  Finland, France (metropolitan), French Guyana, Georgia, Germany, Greece, France, Guadeloupe, Guam, Hungary, Iceland, Ireland, Israel, Italy, Japan, Kazakhstan,  Korea, Republic of, Kuwait, Latvia, Lithuania, Luxembourg, Malaysia, Malta, France, Martinique, Mauritius, Montenegro, Oman, The Netherlands, New Caledonia,  New Zealand, Norway, Panama, Poland, Portugal, Puerto Rico, Qatar, France, La Réunion, Romania, Russian Federation, Saudi Arabia, Serbia, Singapore,  Slovakia, Slovenia, Spain, Sweden, Switzerland, Thailand, Trinidad and Tobago, United Arab Emirates, Türkiye, United Kingdom, United States of America,  Uruguay |
| High HDI | Albania, Algeria, Azerbaijan, Armenia, Barbados, Bosnia Herzegovina, Brazil, Bulgaria, Sri Lanka, China, Colombia, Cuba, Dominican Republic, Ecuador, Fiji,  French Polynesia, Gabon, Gaza Strip and West Bank, Guyana, Indonesia, Iran, Islamic Republic of, Jamaica, Jordan, Lebanon, Libya, Maldives, Mexico, Mongolia,  Moldova, Paraguay, Peru, Saint Lucia, Viet Nam, South Africa, Suriname, Tunisia, Turkmenistan, Ukraine, North Macedonia, Egypt, Uzbekistan, Samoa |
| Medium HDI | Angola, Bangladesh, Bhutan, Bolivia, Botswana, Belize, Solomon Islands, Myanmar, Cambodia, Cameroon, Cape Verde, Comoros, Congo, Republic of, El  Salvador, Equatorial Guinea, Ghana, Guatemala, Honduras, India, Iraq, Côte d'Ivoire, Kenya, Korea, Democratic People Republic of, Kyrgyzstan, Lao People's  Democratic Republic, Mauritania, Morocco, Namibia, Nepal, Vanuatu, Nicaragua, Papua New Guinea, Philippines, Timor-Leste, Sao Tome and Principe,  Zimbabwe, Eswatini, Syrian Arab Republic, Tajikistan, Venezuela, Zambia |
| Low HDI | Afghanistan, Burundi, Central African Republic, Chad, Congo, Democratic People Republic of, Benin, Ethiopia, Eritrea, Djibouti, The Republic of the Gambia,  Guinea, Haiti, Lesotho, Liberia, Madagascar, Malawi, Mali, Mozambique, Niger, Nigeria, Pakistan, Guinea-Bissau, Rwanda, Senegal, Sierra Leone, Somalia, South  Sudan, Sudan, Togo, Uganda, Tanzania, United Republic of, Burkina Faso, Yemen |
| **Note:** HDI = human development index. We used data on HDI for each country from the United Nations website (<https://hdr.undp.org/data-center/human-development-index>). | |

**Table S3. Estimated absolute number, proportion, and ASRs of incidence and mortality by country and sex, aged 15 to 49 years worldwide in 2022.**

| **Country or territory** | **Estimated new cancer cases and ASR per 100 000 people per year** | | | | | | | |  | **Estimated cancer-related deaths and ASR per 100 000 people per year** | | | | | | | |
| --- | --- | --- | --- | --- | --- | --- | --- | --- | --- | --- | --- | --- | --- | --- | --- | --- | --- |
|  | **Both sexes** | |  | Male | |  | Female | |  | **Both sexes** | |  | Male | |  | Female | |
|  | Cases | ASR |  | Cases | ASR |  | Cases | ASR |  | Cases | ASR |  | Cases | ASR |  | Cases | ASR |
| Afghanistan | 246 | 1.51 |  | 153 | 1.8 |  | 93 | 1.19 |  | 263 | 1.62 |  | 179 | 2.11 |  | 84 | 1.09 |
| Albania | 70 | 5.21 |  | 50 | 7.45 |  | 20 | 3.03 |  | 52 | 4.02 |  | 42 | 6.71 |  | 10 | 1.5 |
| Algeria | 537 | 2.3 |  | 419 | 3.61 |  | 118 | 0.98 |  | 403 | 1.83 |  | 317 | 2.92 |  | 86 | 0.73 |
| Angola | 64 | 0.55 |  | 55 | 0.98 |  | 9 | 0.14 |  | 49 | 0.42 |  | 42 | 0.76 |  | 7 | 0.11 |
| Argentina | 543 | 2.38 |  | 334 | 2.99 |  | 209 | 1.79 |  | 408 | 1.68 |  | 241 | 2.01 |  | 167 | 1.36 |
| Armenia | 49 | 3.34 |  | 33 | 5.12 |  | 16 | 1.87 |  | 44 | 3.2 |  | 31 | 5.02 |  | 13 | 1.5 |
| Australia | 351 | 2.62 |  | 162 | 2.36 |  | 189 | 2.88 |  | 175 | 1.26 |  | 94 | 1.35 |  | 81 | 1.17 |
| Austria | 203 | 4.29 |  | 99 | 4.17 |  | 104 | 4.42 |  | 64 | 1.35 |  | 26 | 1.1 |  | 38 | 1.6 |
| Azerbaijan | 235 | 4.37 |  | 189 | 7.16 |  | 46 | 1.68 |  | 194 | 3.6 |  | 158 | 6 |  | 36 | 1.29 |
| Bahamas | 3 | 1.33 |  | 1 | 0.9 |  | 2 | 1.72 |  | 1 | 0.43 |  | 1 | 0.9 |  | 0 | 0 |
| Bahrain | 10 | 0.77 |  | 6 | 0.73 |  | 4 | 0.94 |  | 7 | 0.44 |  | 5 | 0.4 |  | 2 | 0.52 |
| Bangladesh | 1530 | 1.72 |  | 988 | 2.27 |  | 542 | 1.18 |  | 1408 | 1.61 |  | 920 | 2.14 |  | 488 | 1.08 |
| Barbados | 3 | 1.9 |  | 3 | 3.91 |  | 0 | 0 |  | 1 | 0.62 |  | 1 | 1.29 |  | 0 | 0 |
| Belarus | 256 | 4.84 |  | 207 | 8.03 |  | 49 | 1.84 |  | 140 | 2.64 |  | 121 | 4.73 |  | 19 | 0.7 |
| Belgium | 235 | 3.86 |  | 124 | 4.03 |  | 111 | 3.69 |  | 94 | 1.52 |  | 43 | 1.36 |  | 51 | 1.68 |
| Belize | 1 | 0.57 |  | 0 | 0 |  | 1 | 1.09 |  | 1 | 0.57 |  | 0 | 0 |  | 1 | 1.09 |
| Benin | 27 | 0.53 |  | 17 | 0.68 |  | 10 | 0.38 |  | 15 | 0.3 |  | 10 | 0.4 |  | 5 | 0.19 |
| Bhutan | 4 | 1.02 |  | 2 | 0.93 |  | 2 | 1.14 |  | 4 | 1.02 |  | 2 | 0.93 |  | 2 | 1.14 |
| Bolivia | 47 | 0.85 |  | 20 | 0.73 |  | 27 | 0.97 |  | 37 | 0.65 |  | 16 | 0.54 |  | 21 | 0.77 |
| Bosnia Herzegovina | 184 | 10.17 |  | 135 | 14.69 |  | 49 | 5.52 |  | 79 | 4.34 |  | 55 | 5.97 |  | 24 | 2.66 |
| Botswana | 9 | 0.78 |  | 6 | 1.17 |  | 3 | 0.45 |  | 7 | 0.61 |  | 4 | 0.8 |  | 3 | 0.45 |
| Brazil | 2344 | 2.01 |  | 1115 | 1.97 |  | 1229 | 2.04 |  | 1581 | 1.29 |  | 711 | 1.18 |  | 870 | 1.4 |
| Brunei Darussalam | 11 | 3.82 |  | 6 | 3.96 |  | 5 | 3.65 |  | 3 | 1.06 |  | 2 | 1.34 |  | 1 | 0.75 |
| Bulgaria | 231 | 5.5 |  | 143 | 6.65 |  | 88 | 4.28 |  | 186 | 4.34 |  | 113 | 5.1 |  | 73 | 3.55 |
| Burkina Faso | 62 | 0.75 |  | 0 | 0 |  | 62 | 1.49 |  | 59 | 0.72 |  | 0 | 0 |  | 59 | 1.42 |
| Burundi | 40 | 0.91 |  | 17 | 0.74 |  | 23 | 1.06 |  | 32 | 0.73 |  | 13 | 0.56 |  | 19 | 0.88 |
| Cambodia | 342 | 4.7 |  | 218 | 6.39 |  | 124 | 3.2 |  | 292 | 4.12 |  | 193 | 5.76 |  | 99 | 2.66 |
| Cameroon | 69 | 0.67 |  | 44 | 0.89 |  | 25 | 0.45 |  | 49 | 0.51 |  | 31 | 0.67 |  | 18 | 0.35 |
| Canada | 396 | 1.98 |  | 175 | 1.77 |  | 221 | 2.2 |  | 191 | 0.93 |  | 101 | 1 |  | 90 | 0.87 |
| Cape Verde | 1 | 0.23 |  | 0 | 0 |  | 1 | 0.48 |  | 0 | 0 |  | 0 | 0 |  | 0 | 0 |
| Central African Republic | 5 | 0.35 |  | 5 | 0.71 |  | 0 | 0 |  | 3 | 0.23 |  | 3 | 0.47 |  | 0 | 0 |

| Chad | 48 | 0.81 |  | 48 | 1.61 |  | 0 | 0 |  | 46 | 0.73 |  | 46 | 1.47 |  | 0 | 0 |
| --- | --- | --- | --- | --- | --- | --- | --- | --- | --- | --- | --- | --- | --- | --- | --- | --- | --- |
| Chile | 97 | 0.87 |  | 57 | 1 |  | 40 | 0.73 |  | 82 | 0.77 |  | 43 | 0.81 |  | 39 | 0.73 |
| China | 67052 | 8.21 |  | 32094 | 7.67 |  | 34958 | 8.78 |  | 25988 | 3.17 |  | 16843 | 4.01 |  | 9145 | 2.29 |
| Colombia | 277 | 0.96 |  | 142 | 0.97 |  | 135 | 0.94 |  | 226 | 0.8 |  | 98 | 0.69 |  | 128 | 0.89 |
| Comoros | 0 | 0 |  | 0 | 0 |  | 0 | 0 |  | 0 | 0 |  | 0 | 0 |  | 0 | 0 |
| Congo, Democratic People Republic of | 165 | 0.51 |  | 91 | 0.58 |  | 74 | 0.44 |  | 133 | 0.42 |  | 75 | 0.49 |  | 58 | 0.36 |
| Congo, Republic of | 4 | 0.18 |  | 3 | 0.27 |  | 1 | 0.09 |  | 1 | 0.05 |  | 1 | 0.09 |  | 0 | 0 |
| Costa Rica | 24 | 0.83 |  | 14 | 0.97 |  | 10 | 0.7 |  | 17 | 0.59 |  | 8 | 0.55 |  | 9 | 0.62 |
| Croatia | 56 | 2.49 |  | 25 | 2.21 |  | 31 | 2.78 |  | 55 | 2.46 |  | 35 | 3.11 |  | 20 | 1.8 |
| Cuba | 153 | 2.57 |  | 85 | 2.61 |  | 68 | 2.53 |  | 98 | 1.54 |  | 50 | 1.57 |  | 48 | 1.52 |
| Cyprus | 27 | 3.83 |  | 17 | 4.92 |  | 10 | 2.79 |  | 10 | 1.43 |  | 5 | 1.43 |  | 5 | 1.42 |
| Czechia | 146 | 2.09 |  | 77 | 2.12 |  | 69 | 2.06 |  | 112 | 1.5 |  | 60 | 1.57 |  | 52 | 1.44 |
| Côte d'Ivoire | 86 | 0.87 |  | 56 | 1.14 |  | 30 | 0.6 |  | 58 | 0.6 |  | 40 | 0.83 |  | 18 | 0.37 |
| Denmark | 91 | 3.15 |  | 41 | 2.83 |  | 50 | 3.48 |  | 55 | 1.85 |  | 19 | 1.28 |  | 36 | 2.42 |
| Djibouti | 6 | 1.12 |  | 4 | 1.38 |  | 2 | 0.81 |  | 3 | 0.63 |  | 2 | 0.77 |  | 1 | 0.45 |
| Dominican Republic | 102 | 1.89 |  | 52 | 1.93 |  | 50 | 1.84 |  | 111 | 2.01 |  | 60 | 2.2 |  | 51 | 1.83 |
| Ecuador | 93 | 1.01 |  | 40 | 0.88 |  | 53 | 1.14 |  | 79 | 0.85 |  | 35 | 0.75 |  | 44 | 0.95 |
| Egypt | 1025 | 2.09 |  | 667 | 2.73 |  | 358 | 1.43 |  | 756 | 1.56 |  | 483 | 2.01 |  | 273 | 1.1 |
| El Salvador | 38 | 1.18 |  | 16 | 1.1 |  | 22 | 1.24 |  | 30 | 0.93 |  | 16 | 1.1 |  | 14 | 0.78 |
| Equatorial Guinea | 6 | 0.83 |  | 3 | 0.91 |  | 3 | 0.79 |  | 5 | 0.71 |  | 2 | 0.71 |  | 3 | 0.79 |
| Eritrea | 12 | 0.94 |  | 8 | 1.29 |  | 4 | 0.61 |  | 6 | 0.46 |  | 4 | 0.64 |  | 2 | 0.28 |
| Estonia | 16 | 2.11 |  | 14 | 3.62 |  | 2 | 0.53 |  | 9 | 1.16 |  | 8 | 2.02 |  | 1 | 0.26 |
| Eswatini | 8 | 1.47 |  | 4 | 1.6 |  | 4 | 1.36 |  | 8 | 1.47 |  | 4 | 1.6 |  | 4 | 1.36 |
| Ethiopia | 529 | 1.17 |  | 275 | 1.2 |  | 254 | 1.14 |  | 523 | 1.17 |  | 271 | 1.2 |  | 252 | 1.14 |
| Fiji | 9 | 1.97 |  | 6 | 2.51 |  | 3 | 1.4 |  | 9 | 1.96 |  | 6 | 2.49 |  | 3 | 1.4 |
| Finland | 48 | 1.8 |  | 32 | 2.33 |  | 16 | 1.24 |  | 13 | 0.5 |  | 9 | 0.67 |  | 4 | 0.31 |
| France (metropolitan) | 2367 | 7.06 |  | 1323 | 7.96 |  | 1044 | 6.18 |  | 1316 | 3.88 |  | 796 | 4.75 |  | 520 | 3.03 |
| France, Guadeloupe | 6 | 3.21 |  | 3 | 3.93 |  | 3 | 2.72 |  | 5 | 2.58 |  | 2 | 2.32 |  | 3 | 2.72 |
| France, La Réunion | 17 | 3.68 |  | 11 | 5.04 |  | 6 | 2.46 |  | 10 | 2.17 |  | 4 | 1.82 |  | 6 | 2.46 |
| France, Martinique | 2 | 1.09 |  | 1 | 1.31 |  | 1 | 0.93 |  | 2 | 1.09 |  | 1 | 1.31 |  | 1 | 0.93 |
| French Guyana | 4 | 2.6 |  | 3 | 3.94 |  | 1 | 1.32 |  | 4 | 2.6 |  | 3 | 3.94 |  | 1 | 1.32 |
| French Polynesia | 9 | 5.6 |  | 6 | 7.4 |  | 3 | 3.77 |  | 5 | 3.08 |  | 4 | 4.9 |  | 1 | 1.24 |
| Gabon | 9 | 0.82 |  | 4 | 0.74 |  | 5 | 0.89 |  | 9 | 0.82 |  | 4 | 0.74 |  | 5 | 0.89 |
| Gaza Strip and West Bank | 59 | 2.8 |  | 43 | 4.09 |  | 16 | 1.48 |  | 53 | 2.57 |  | 42 | 4.04 |  | 11 | 1.06 |
| Georgia | 89 | 4.53 |  | 73 | 7.66 |  | 16 | 1.49 |  | 66 | 3.28 |  | 55 | 5.62 |  | 11 | 1.05 |

| Germany | 1012 | 2.5 |  | 545 | 2.68 |  | 467 | 2.32 |  | 725 | 1.78 |  | 405 | 1.98 |  | 320 | 1.58 |
| --- | --- | --- | --- | --- | --- | --- | --- | --- | --- | --- | --- | --- | --- | --- | --- | --- | --- |
| Ghana | 124 | 0.79 |  | 41 | 0.54 |  | 83 | 1.04 |  | 110 | 0.72 |  | 39 | 0.53 |  | 71 | 0.92 |
| Greece | 118 | 1.84 |  | 65 | 2.02 |  | 53 | 1.67 |  | 85 | 1.33 |  | 51 | 1.58 |  | 34 | 1.07 |
| Guam | 2 | 2.47 |  | 1 | 2.45 |  | 1 | 2.48 |  | 0 | 0 |  | 0 | 0 |  | 0 | 0 |
| Guatemala | 78 | 0.92 |  | 41 | 1.01 |  | 37 | 0.83 |  | 74 | 0.88 |  | 40 | 1 |  | 34 | 0.77 |
| Guinea | 103 | 2.32 |  | 72 | 3.62 |  | 31 | 1.29 |  | 109 | 2.44 |  | 75 | 3.7 |  | 34 | 1.43 |
| Guinea-Bissau | 7 | 0.93 |  | 5 | 1.35 |  | 2 | 0.55 |  | 7 | 0.93 |  | 5 | 1.35 |  | 2 | 0.55 |
| Guyana | 3 | 0.83 |  | 3 | 1.66 |  | 0 | 0 |  | 4 | 1.03 |  | 4 | 2.06 |  | 0 | 0 |
| Haiti | 86 | 1.67 |  | 29 | 1.14 |  | 57 | 2.16 |  | 48 | 0.87 |  | 25 | 1.03 |  | 23 | 0.74 |
| Honduras | 50 | 1 |  | 24 | 0.98 |  | 26 | 1.01 |  | 34 | 0.68 |  | 13 | 0.54 |  | 21 | 0.83 |
| Hungary | 338 | 5.14 |  | 199 | 6.03 |  | 139 | 4.24 |  | 244 | 3.71 |  | 147 | 4.46 |  | 97 | 2.96 |
| Iceland | 3 | 1.69 |  | 1 | 1.14 |  | 2 | 2.26 |  | 1 | 0.57 |  | 1 | 1.14 |  | 0 | 0 |
| India | 8937 | 1.21 |  | 5303 | 1.4 |  | 3634 | 1 |  | 7756 | 1.08 |  | 4602 | 1.26 |  | 3154 | 0.88 |
| Indonesia | 4821 | 3.06 |  | 3678 | 4.65 |  | 1143 | 1.46 |  | 2893 | 1.84 |  | 2181 | 2.77 |  | 712 | 0.91 |
| Iran, Islamic Republic of | 1385 | 2.77 |  | 822 | 3.35 |  | 563 | 2.19 |  | 919 | 1.88 |  | 574 | 2.41 |  | 345 | 1.34 |
| Iraq | 233 | 1.2 |  | 185 | 1.87 |  | 48 | 0.52 |  | 196 | 1.04 |  | 159 | 1.66 |  | 37 | 0.4 |
| Ireland | 77 | 2.67 |  | 31 | 2.14 |  | 46 | 3.2 |  | 24 | 0.76 |  | 17 | 1.1 |  | 7 | 0.44 |
| Israel | 114 | 2.73 |  | 71 | 3.41 |  | 43 | 2.05 |  | 58 | 1.34 |  | 44 | 2.04 |  | 14 | 0.64 |
| Italy | 778 | 2.24 |  | 393 | 2.31 |  | 385 | 2.17 |  | 388 | 1.06 |  | 184 | 1.01 |  | 204 | 1.1 |
| Jamaica | 34 | 2.25 |  | 19 | 2.66 |  | 15 | 1.9 |  | 27 | 1.8 |  | 16 | 2.25 |  | 11 | 1.41 |
| Japan | 2166 | 3.39 |  | 1091 | 3.59 |  | 1075 | 3.17 |  | 345 | 0.56 |  | 184 | 0.61 |  | 161 | 0.5 |
| Jordan | 179 | 3.66 |  | 140 | 5.63 |  | 39 | 1.58 |  | 136 | 2.79 |  | 107 | 4.31 |  | 29 | 1.18 |
| Kazakhstan | 267 | 2.91 |  | 212 | 4.8 |  | 55 | 1.09 |  | 215 | 2.34 |  | 166 | 3.76 |  | 49 | 0.97 |
| Kenya | 118 | 0.51 |  | 45 | 0.42 |  | 73 | 0.6 |  | 83 | 0.37 |  | 30 | 0.29 |  | 53 | 0.45 |
| Korea, Democratic People Republic of | 949 | 6.59 |  | 589 | 8.14 |  | 360 | 5.02 |  | 613 | 4.53 |  | 400 | 5.86 |  | 213 | 3.16 |
| Korea, Republic of | 1072 | 3.48 |  | 505 | 3.24 |  | 567 | 3.72 |  | 263 | 0.88 |  | 158 | 1.04 |  | 105 | 0.68 |
| Kuwait | 15 | 0.4 |  | 9 | 0.38 |  | 6 | 0.44 |  | 7 | 0.27 |  | 5 | 0.32 |  | 2 | 0.18 |
| Kyrgyzstan | 49 | 1.6 |  | 30 | 1.98 |  | 19 | 1.21 |  | 43 | 1.3 |  | 25 | 1.43 |  | 18 | 1.17 |
| Lao People's Democratic Republic | 158 | 4.57 |  | 98 | 5.82 |  | 60 | 3.32 |  | 141 | 4.14 |  | 96 | 5.71 |  | 45 | 2.58 |
| Latvia | 23 | 2.34 |  | 13 | 2.69 |  | 10 | 1.94 |  | 23 | 2.23 |  | 18 | 3.58 |  | 5 | 0.96 |
| Lebanon | 144 | 4.14 |  | 78 | 4.55 |  | 66 | 3.73 |  | 117 | 3.38 |  | 65 | 3.8 |  | 52 | 2.95 |
| Lesotho | 7 | 0.73 |  | 4 | 0.84 |  | 3 | 0.63 |  | 4 | 0.47 |  | 3 | 0.67 |  | 1 | 0.25 |
| Liberia | 33 | 1.6 |  | 25 | 2.38 |  | 8 | 0.82 |  | 30 | 1.5 |  | 22 | 2.17 |  | 8 | 0.82 |
| Libya | 125 | 2.84 |  | 107 | 4.79 |  | 18 | 0.82 |  | 106 | 2.4 |  | 91 | 4.08 |  | 15 | 0.68 |
| Lithuania | 60 | 4.34 |  | 40 | 5.78 |  | 20 | 3 |  | 38 | 2.66 |  | 30 | 4.27 |  | 8 | 1.14 |

| Luxembourg | 7 | 1.81 |  | 3 | 1.49 |  | 4 | 2.16 |  | 8 | 2.06 |  | 4 | 1.98 |  | 4 | 2.16 |
| --- | --- | --- | --- | --- | --- | --- | --- | --- | --- | --- | --- | --- | --- | --- | --- | --- | --- |
| Madagascar | 68 | 0.6 |  | 24 | 0.43 |  | 44 | 0.77 |  | 55 | 0.51 |  | 20 | 0.36 |  | 35 | 0.65 |
| Malawi | 26 | 0.36 |  | 18 | 0.58 |  | 8 | 0.15 |  | 23 | 0.32 |  | 15 | 0.5 |  | 8 | 0.15 |
| Malaysia | 615 | 3.42 |  | 474 | 5.06 |  | 141 | 1.63 |  | 389 | 2.15 |  | 285 | 3.03 |  | 104 | 1.19 |
| Maldives | 8 | 2.28 |  | 8 | 3.55 |  | 0 | 0 |  | 4 | 1.26 |  | 4 | 1.99 |  | 0 | 0 |
| Mali | 69 | 0.97 |  | 45 | 1.3 |  | 24 | 0.65 |  | 71 | 1.01 |  | 46 | 1.34 |  | 25 | 0.69 |
| Malta | 4 | 1.62 |  | 1 | 0.8 |  | 3 | 2.49 |  | 4 | 1.62 |  | 1 | 0.8 |  | 3 | 2.49 |
| Mauritania | 14 | 0.71 |  | 10 | 1 |  | 4 | 0.42 |  | 11 | 0.56 |  | 8 | 0.82 |  | 3 | 0.3 |
| Mauritius | 12 | 1.61 |  | 7 | 1.87 |  | 5 | 1.34 |  | 8 | 1.07 |  | 5 | 1.34 |  | 3 | 0.81 |
| Mexico | 494 | 0.69 |  | 240 | 0.68 |  | 254 | 0.69 |  | 506 | 0.71 |  | 268 | 0.78 |  | 238 | 0.65 |
| Moldova | 126 | 5.28 |  | 97 | 8.46 |  | 29 | 2.26 |  | 84 | 3.58 |  | 63 | 5.63 |  | 21 | 1.65 |
| Mongolia | 38 | 2.03 |  | 29 | 3.15 |  | 9 | 0.96 |  | 25 | 1.49 |  | 18 | 2.24 |  | 7 | 0.76 |
| Montenegro | 24 | 6.95 |  | 17 | 10.04 |  | 7 | 3.97 |  | 16 | 4.64 |  | 10 | 5.96 |  | 6 | 3.4 |
| Morocco | 831 | 4.43 |  | 698 | 7.93 |  | 133 | 1.32 |  | 832 | 4.45 |  | 720 | 8.13 |  | 112 | 1.13 |
| Mozambique | 36 | 0.28 |  | 24 | 0.45 |  | 12 | 0.14 |  | 30 | 0.25 |  | 21 | 0.43 |  | 9 | 0.1 |
| Myanmar | 838 | 2.83 |  | 504 | 3.56 |  | 334 | 2.17 |  | 631 | 2.1 |  | 399 | 2.79 |  | 232 | 1.47 |
| Namibia | 14 | 1.3 |  | 8 | 1.51 |  | 6 | 1.1 |  | 8 | 0.79 |  | 4 | 0.8 |  | 4 | 0.79 |
| Nepal | 244 | 1.76 |  | 145 | 2.6 |  | 99 | 1.18 |  | 220 | 1.61 |  | 137 | 2.49 |  | 83 | 1.02 |
| New Caledonia | 14 | 8.52 |  | 8 | 9.8 |  | 6 | 7.18 |  | 9 | 5.47 |  | 5 | 6.16 |  | 4 | 4.77 |
| New Zealand | 78 | 3.21 |  | 32 | 2.62 |  | 46 | 3.74 |  | 55 | 2.29 |  | 24 | 2.04 |  | 31 | 2.54 |
| Nicaragua | 30 | 0.89 |  | 15 | 0.91 |  | 15 | 0.85 |  | 30 | 0.86 |  | 13 | 0.8 |  | 17 | 0.91 |
| Niger | 16 | 0.21 |  | 16 | 0.45 |  | 0 | 0 |  | 15 | 0.19 |  | 15 | 0.41 |  | 0 | 0 |
| Nigeria | 181 | 0.24 |  | 63 | 0.16 |  | 118 | 0.31 |  | 166 | 0.22 |  | 64 | 0.16 |  | 102 | 0.27 |
| North Macedonia | 78 | 6.33 |  | 59 | 9.41 |  | 19 | 3.14 |  | 57 | 4.66 |  | 43 | 6.93 |  | 14 | 2.31 |
| Norway | 62 | 2.12 |  | 25 | 1.65 |  | 37 | 2.61 |  | 3 | 0.1 |  | 2 | 0.13 |  | 1 | 0.07 |
| Oman | 27 | 0.73 |  | 21 | 0.78 |  | 6 | 0.6 |  | 23 | 0.67 |  | 19 | 0.76 |  | 4 | 0.43 |
| Pakistan | 1156 | 1.15 |  | 733 | 1.45 |  | 423 | 0.84 |  | 977 | 1.01 |  | 601 | 1.24 |  | 376 | 0.77 |
| Panama | 14 | 0.59 |  | 6 | 0.51 |  | 8 | 0.68 |  | 11 | 0.47 |  | 4 | 0.36 |  | 7 | 0.59 |
| Papua New Guinea | 85 | 2.03 |  | 46 | 2.13 |  | 39 | 1.92 |  | 59 | 1.51 |  | 32 | 1.63 |  | 27 | 1.39 |
| Paraguay | 43 | 1.22 |  | 27 | 1.51 |  | 16 | 0.93 |  | 35 | 0.93 |  | 22 | 1.1 |  | 13 | 0.76 |
| Peru | 299 | 1.64 |  | 141 | 1.52 |  | 158 | 1.76 |  | 261 | 1.41 |  | 129 | 1.39 |  | 132 | 1.44 |
| Philippines | 2309 | 4.34 |  | 1527 | 5.74 |  | 782 | 2.91 |  | 1651 | 3.15 |  | 1102 | 4.23 |  | 549 | 2.07 |
| Poland | 584 | 2.63 |  | 364 | 3.27 |  | 220 | 1.99 |  | 411 | 1.85 |  | 272 | 2.44 |  | 139 | 1.26 |
| Portugal | 279 | 4.59 |  | 148 | 4.91 |  | 131 | 4.31 |  | 164 | 2.57 |  | 96 | 3.08 |  | 68 | 2.1 |
| Puerto Rico | 23 | 1.51 |  | 13 | 1.81 |  | 10 | 1.22 |  | 11 | 0.74 |  | 7 | 1.01 |  | 4 | 0.48 |

| Qatar | 20 | 0.89 |  | 18 | 1.03 |  | 2 | 0.32 |  | 14 | 0.66 |  | 12 | 0.75 |  | 2 | 0.32 |
| --- | --- | --- | --- | --- | --- | --- | --- | --- | --- | --- | --- | --- | --- | --- | --- | --- | --- |
| Romania | 823 | 6.97 |  | 559 | 9.3 |  | 264 | 4.59 |  | 589 | 4.9 |  | 395 | 6.42 |  | 194 | 3.36 |
| Russia | 3396 | 4.05 |  | 2303 | 5.7 |  | 1093 | 2.52 |  | 1953 | 2.3 |  | 1374 | 3.34 |  | 579 | 1.33 |
| Rwanda | 58 | 1 |  | 48 | 1.74 |  | 10 | 0.31 |  | 46 | 0.8 |  | 38 | 1.39 |  | 8 | 0.25 |
| Saint Lucia | 1 | 0.98 |  | 1 | 2.03 |  | 0 | 0 |  | 1 | 0.98 |  | 1 | 2.03 |  | 0 | 0 |
| Samoa | 4 | 5.09 |  | 3 | 7.35 |  | 1 | 2.68 |  | 4 | 5.09 |  | 3 | 7.35 |  | 1 | 2.68 |
| Sao Tome and Principe | 2 | 2.21 |  | 2 | 4.44 |  | 0 | 0 |  | 2 | 2.21 |  | 2 | 4.44 |  | 0 | 0 |
| Saudi Arabia | 231 | 0.9 |  | 159 | 0.98 |  | 72 | 0.75 |  | 137 | 0.54 |  | 94 | 0.59 |  | 43 | 0.46 |
| Senegal | 86 | 1.35 |  | 59 | 2.02 |  | 27 | 0.79 |  | 75 | 1.19 |  | 52 | 1.79 |  | 23 | 0.69 |
| Serbia | 306 | 6.12 |  | 187 | 7.52 |  | 119 | 4.76 |  | 193 | 3.85 |  | 121 | 4.87 |  | 72 | 2.86 |
| Sierra Leone | 10 | 0.23 |  | 9 | 0.4 |  | 1 | 0.05 |  | 6 | 0.13 |  | 5 | 0.21 |  | 1 | 0.05 |
| Singapore | 131 | 3.45 |  | 68 | 3.44 |  | 63 | 3.43 |  | 70 | 1.87 |  | 40 | 2.05 |  | 30 | 1.66 |
| Slovakia | 43 | 1.23 |  | 20 | 1.12 |  | 23 | 1.34 |  | 37 | 1.06 |  | 20 | 1.12 |  | 17 | 0.99 |
| Slovenia | 29 | 2.36 |  | 16 | 2.48 |  | 13 | 2.24 |  | 32 | 2.59 |  | 22 | 3.37 |  | 10 | 1.72 |
| Solomon Islands | 6 | 1.88 |  | 5 | 3.06 |  | 1 | 0.71 |  | 3 | 0.99 |  | 2 | 1.27 |  | 1 | 0.71 |
| Somalia | 45 | 0.87 |  | 23 | 0.9 |  | 22 | 0.85 |  | 35 | 0.7 |  | 18 | 0.72 |  | 17 | 0.68 |
| South Africa | 900 | 2.82 |  | 579 | 3.66 |  | 321 | 2.01 |  | 686 | 2.32 |  | 472 | 3.3 |  | 214 | 1.37 |
| South Sudan | 21 | 0.49 |  | 12 | 0.58 |  | 9 | 0.41 |  | 14 | 0.32 |  | 8 | 0.38 |  | 6 | 0.27 |
| Spain | 1138 | 3.61 |  | 463 | 2.79 |  | 675 | 4.43 |  | 655 | 2.01 |  | 328 | 1.96 |  | 327 | 2.05 |
| Sri Lanka | 160 | 1.39 |  | 96 | 1.75 |  | 64 | 1.06 |  | 146 | 1.28 |  | 89 | 1.64 |  | 57 | 0.95 |
| Sudan | 96 | 0.57 |  | 76 | 0.95 |  | 20 | 0.21 |  | 86 | 0.52 |  | 72 | 0.92 |  | 14 | 0.15 |
| Suriname | 12 | 4.01 |  | 10 | 6.73 |  | 2 | 1.33 |  | 8 | 2.7 |  | 7 | 4.77 |  | 1 | 0.66 |
| Sweden | 92 | 1.8 |  | 33 | 1.27 |  | 59 | 2.35 |  | 13 | 0.25 |  | 8 | 0.3 |  | 5 | 0.2 |
| Switzerland | 73 | 1.55 |  | 43 | 1.81 |  | 30 | 1.28 |  | 38 | 0.81 |  | 24 | 1.01 |  | 14 | 0.6 |
| Syrian Arab Republic | 324 | 3.5 |  | 230 | 5.15 |  | 94 | 1.94 |  | 311 | 3.41 |  | 211 | 4.87 |  | 100 | 2.02 |
| Tajikistan | 72 | 1.66 |  | 47 | 2.18 |  | 25 | 1.14 |  | 60 | 1.34 |  | 38 | 1.74 |  | 22 | 0.95 |
| Tanzania, United Republic of | 220 | 0.93 |  | 112 | 0.92 |  | 108 | 0.93 |  | 173 | 0.74 |  | 89 | 0.76 |  | 84 | 0.73 |
| Thailand | 1561 | 3.8 |  | 934 | 4.66 |  | 627 | 3 |  | 857 | 2.04 |  | 500 | 2.44 |  | 357 | 1.67 |
| The Netherlands | 342 | 4 |  | 141 | 3.29 |  | 201 | 4.7 |  | 139 | 1.59 |  | 71 | 1.64 |  | 68 | 1.54 |
| The Republic of the Gambia | 10 | 1.26 |  | 9 | 2.33 |  | 1 | 0.28 |  | 11 | 1.36 |  | 10 | 2.52 |  | 1 | 0.28 |
| Timor-Leste | 9 | 1.99 |  | 7 | 2.99 |  | 2 | 0.97 |  | 6 | 1.37 |  | 4 | 1.76 |  | 2 | 0.97 |
| Togo | 23 | 0.65 |  | 18 | 1.02 |  | 5 | 0.29 |  | 17 | 0.47 |  | 14 | 0.77 |  | 3 | 0.17 |
| Trinidad and Tobago | 15 | 1.94 |  | 12 | 3.16 |  | 3 | 0.74 |  | 10 | 1.26 |  | 8 | 2.04 |  | 2 | 0.48 |
| Tunisia | 361 | 5.41 |  | 295 | 9.14 |  | 66 | 1.89 |  | 292 | 4.49 |  | 246 | 7.82 |  | 46 | 1.32 |
| Turkmenistan | 57 | 1.89 |  | 36 | 2.51 |  | 21 | 1.32 |  | 52 | 1.75 |  | 35 | 2.48 |  | 17 | 1.07 |
| Türkiye | 3386 | 7.28 |  | 2385 | 10.48 |  | 1001 | 4.17 |  | 1369 | 3.09 |  | 966 | 4.5 |  | 403 | 1.68 |
| Uganda | 160 | 0.96 |  | 91 | 1.14 |  | 69 | 0.8 |  | 109 | 0.65 |  | 62 | 0.78 |  | 47 | 0.54 |
| Ukraine | 1054 | 4 |  | 787 | 6.14 |  | 267 | 1.99 |  | 789 | 2.99 |  | 610 | 4.77 |  | 179 | 1.32 |
| United Arab Emirates | 24 | 0.29 |  | 11 | 0.19 |  | 13 | 0.59 |  | 21 | 0.21 |  | 15 | 0.19 |  | 6 | 0.28 |
| United Kingdom | 981 | 2.8 |  | 510 | 2.92 |  | 471 | 2.68 |  | 295 | 0.84 |  | 161 | 0.93 |  | 134 | 0.76 |
| USA | 4493 | 2.7 |  | 2070 | 2.48 |  | 2423 | 2.91 |  | 1453 | 0.87 |  | 727 | 0.86 |  | 726 | 0.87 |
| Uruguay | 93 | 5.08 |  | 57 | 6.33 |  | 36 | 3.85 |  | 53 | 2.82 |  | 34 | 3.62 |  | 19 | 2.01 |
| Uzbekistan | 268 | 1.54 |  | 140 | 1.66 |  | 128 | 1.42 |  | 227 | 1.24 |  | 117 | 1.28 |  | 110 | 1.2 |
| Vanuatu | 2 | 1.43 |  | 2 | 2.8 |  | 0 | 0 |  | 2 | 1.43 |  | 2 | 2.8 |  | 0 | 0 |
| Venezuela | 336 | 2.32 |  | 194 | 2.73 |  | 142 | 1.92 |  | 247 | 1.7 |  | 135 | 1.95 |  | 112 | 1.46 |
| Viet Nam | 2513 | 4.66 |  | 1618 | 6.1 |  | 895 | 3.22 |  | 2380 | 4.46 |  | 1653 | 6.33 |  | 727 | 2.56 |
| Yemen | 373 | 2.83 |  | 200 | 3.04 |  | 173 | 2.6 |  | 364 | 2.8 |  | 201 | 3.06 |  | 163 | 2.53 |
| Zambia | 63 | 0.96 |  | 28 | 0.85 |  | 35 | 1.06 |  | 41 | 0.6 |  | 17 | 0.48 |  | 24 | 0.71 |
| Zimbabwe | 44 | 0.73 |  | 24 | 0.89 |  | 20 | 0.58 |  | 30 | 0.48 |  | 17 | 0.61 |  | 13 | 0.36 |
| **Note:** ASRs, age-standardized rates; EOTBL, early-onset tracheal, bronchus, and lung | | | | | | | | | | | | | | | | | |

**Table S4. Estimated absolute numbers, proportions, and ASRs of EOTBL cancer incidence and mortality by HDI and sex among individuals aged 15 to 49 years worldwide in 2022.**

| **HDI category** | **Estimated new cancer cases andASR per 100 000 people per year** | | | | | | | |  | **Estimated cancer-related deaths and ASR per 100 000 people per year** | | | | | | | |
| --- | --- | --- | --- | --- | --- | --- | --- | --- | --- | --- | --- | --- | --- | --- | --- | --- | --- |
|  | **Both sexes** | |  | Male | |  | Female | |  | **Both sexes** | |  | Male | |  | Female | |
|  | **Cases** | **ASR** |  | **Cases** | **ASR** |  | **Cases** | **ASR** |  | **Deaths** | **ASR** |  | **Deaths** | **ASR** |  | **Deaths** | **ASR** |
| Very High HDI | 29963 | 3.4 |  | 17089 | 3.8 |  | 12874 | 2.9 |  | 14295 | 1.6 |  | 8746 | 1.9 |  | 5549 | 1.2 |
| High HDI | 85548 | 5.5 |  | 44239 | 5.7 |  | 41309 | 5.3 |  | 39633 | 2.6 |  | 25871 | 3.3 |  | 13762 | 1.8 |
| Medium HDI | 18128 | 1.6 |  | 11263 | 2 |  | 6865 | 1.2 |  | 15143 | 1.4 |  | 9518 | 1.7 |  | 5625 | 1 |
| Low HDI | 4030 | 0.88 |  | 2333 | 1 |  | 1697 | 0.73 |  | 3554 | 0.78 |  | 2084 | 0.93 |  | 1470 | 0.64 |
| **Note:** ASR, age-standardized rate; HDI, Human Development Index; EOTBL, early-onset Tracheal, bronchus, and lung. | | | | | | | | | | | | | | | | | |

**Table S5. Changes in the incidence and mortality of EOTBL cancer across the eight supplementary countries, 1990–2023**

**and 2019–2023.**

| **Location** | **AAPC of incidence rate, % (95% CI)** | | **AAPC of mortality rate, % (95% CI)** | |
| --- | --- | --- | --- | --- |
|  | 1990-2023 | 2019-2023 | 1990-2023 | 2019-2023 |
| **Both** | | | | |
| Bangladesh | 0.78 (0.59 to 0.94) | 9.781 (7.91 to 11.16) | 0.71 (0.52 to 0.86) | 9.66 (7.88 to 11.00) |
| Egypt | 1.97 (1.81 to 2.08) | 7.295 (5.853to 8.28) | 1.86 (1.69 to 1.97) | 7.02 (5.50 to 8.01) |
| Ethiopia | 1.21 (1.12 to 1.30) | 9.234 (8.47 to 9.89) | 1.19 (1.10 to 1.29) | 9.17 (8.37 to 9.86) |
| Indonesia | 2.12 (2.00 to 2.24) | 0.728 (-0.08 to 1.51) | 2.07 (1.96 to 2.19) | 0.67 (-0.12 to 1.46) |
| Japan | -1.57 (-1.73 to -1.46) | -0.973 (-2.154to -0.07) | -2.30 (-2.47 to -2.18) | -0.75 (-2.07 to 0.30) |
| Pakistan | 1.24 (1.09 to 1.42) | 4.451 (2.75 to 5.94) | 1.18 (1.03 to 1.36) | 4.28 (2.54 to 5.75) |
| Papua New Guinea | 1.14 (1.01 to 1.35) | 1.173 (0.94 to 1.85) | 1.10 (0.96 to 1.31) | 1.12 (0.88 to 1.93) |
| UK | -1.99 (-2.18 to -1.90) | -0.596 (-2.31 to 0.52) | -2.35 (-2.55 to -2.67) | -0.6 (-2.42 to 0.2) |
| **Male** | | | | |
| Bangladesh | 0.63 (0.34 to 0.79) | 8.57 (5.97 to 10.01) | 0.57 (0.27 to 0.73) | 8.43 (5.55 to 9.90) |
| Egypt | 1.65 (1.51 to 1.77) | 6.18 (4.94 to 7.18) | 1.57 (1.43 to 1.68) | 5.94 (4.76 to 6.91) |
| Ethiopia | 0.78 (0.68 to 0.86) | 8.42 (7.52 to 9.09) | 0.76 (0.66 to 0.83) | 8.32 (7.54 to 8.94) |
| Indonesia | 2.34 (2.24 to 2.44) | -0.12 (-0.89 to 0.52) | 2.29 (2.20 to 2.39) | -0.19 (-0.92 to 0.47) |
| Japan | -2.06 (-2.23 to -1.89) | -3.10 (-4.59 to -1.37) | -2.71 (-2.85 to -2.55) | -2.69 (-4.00 to -1.10) |
| Pakistan | 0.20 (0.09 to 0.30) | 3.64 (2.25 to 4.45) | 0.17 (0.07 to 0.28) | 3.58 (2.26 to 4.43) |
| Papua New Guinea | 1.03 (0.85 to 1.26) | 0.72 (0.46 to 0.85) | 1.00 (0.83 to 1.23) | 0.68 (0.43 to 0.81) |
| UK | -2.83 (-2.93 to -2.75) | -2.50 (-2.66 to -2.22) | -3.14 (-3.24 to -3.06)) | -2.63 (-2.82 to -2.36) |
| **Female** | | | | |
| Bangladesh | 1.73 (1.52 to 1.93) | 12.27 (10.20 to 14.10) | 1.63 (1.42 to 1.84) | 12.07 (9.89 to 13.86) |
| Egypt | 2.44 (2.30 to 2.56) | 9.25 (8.10 to 10.24) | 2.31 (2.18 to 2.44) | 8.96 (7.86 to 9.98) |
| Ethiopia | 3.12 (2.99 to 3.24) | 12.57 (11.69 to 13.39) | 2.97 (2.80 to 3.09) | 12.45 (10.95 to 13.63) |
| Indonesia | 1.99 (1.82 to 2.15) | 3.23 (2.90 to 3.65) | 1.93 (1.77 to 2.09) | 3.17 (2.84 to 3.57) |
| Japan | -0.93 (-1.07 to -0.83) | 1.66 (0.53 to 2.49) | -1.65 (-1.76 to -1.55) | 1.77 (0.97 to 2.48) |
| Pakistan | 1.95 (1.75 to 2.13) | 2.55 (0.90 to 3.88) | 1.93 (1.72 to 2.12) | 2.41 (0.72 to 3.82) |
| Papua New Guinea | 1.59 (1.39 to 1.75) | 4.68 (3.03 to 5.90) | 1.54 (1.35 to 1.69) | 4.63 (3.09 to 5.79) |
| UK | -0.98 (-1.10 to -0.89) | 0.24 (-0.66 to 1.01) | -1.38 (-1.50 to -1.30) | 0.12 (-0.80 to 0.80) |

Note: AAPC, average annual percent change; CI, confidence interval.

**Table S6. Incidence, mortality, and DALYs of EOTBL cancer among individuals aged 15–49 years across eight selected major countries in 1990 and 2023.**

| **Location** | **Incidence** | | **Mortality** | | **DALYs** | |
| --- | --- | --- | --- | --- | --- | --- |
|  | **ASR per 100,000 (95% UI)** | | **ASR, per 100,000 (95% UI)** | | **ASR, per 100,000 (95% UI)** | |
|  | 1990 | 2023 | 1990 | 2023 | 1990 | 2023 |
| **Both** | | | | | | |
| Australia | 5.25  (4.74 to 5.76) | 3.01  (2.66 to 3.43) | 3.36  (3.09 to 3.64) | 1.51  (1.35 to 1.69) | 155.44  (142.94 to 168.99) | 71.21  (63.63 to 79.89) |
| Brazil | 3.09  (2.88 to 3.35) | 2.15  (1.99 to 2.33) | 2.59  (2.41 to 2.8) | 1.74  (1.61 to 1.88) | 123.09  (114.46 to 133.3) | 84.62  (77.95 to 91.44) |
| China | 7.61  (6.05 to 9.56) | 4.8  (3.75 to 5.96) | 6.33  (5.02 to 7.97) | 3.68  (2.87 to 4.59) | 309.03  (244.64 to 388.92) | 175.53  (137.05 to 219.05) |
| Germany | 7.35  (6.85 to 7.92) | 3.76  (3.32 to 4.29) | 5.75  (5.36 to 6.15) | 2.44  (2.19 to 2.73) | 266.13  (247.72 to 285.2) | 113.58  (101.83 to 127.47) |
| India | 1.29  (0.94 to 1.8) | 1.91  (1.34 to 2.61) | 1.09  (0.8 to 1.52) | 1.58  (1.12 to 2.16) | 52.28  (38.4 to 73.09) | 76.16  (53.6 to 104.23) |
| Nigeria | 1.37  (0.92 to 2.03) | 1.34  (0.91 to 1.96) | 1.15  (0.77 to 1.71) | 1.12  (0.76 to 1.63) | 56.12  (37.76 to 83.34) | 54.67  (37.03 to 79.49) |
| Russia | 8.75  (8 to 9.75) | 3.1  (2.77 to 3.47) | 7.31  (6.68 to 8.16) | 2.45  (2.2 to 2.73) | 337.99  (308.2 to 377.91) | 114.86  (103.09 to 128.51) |
| USA | 8.41  (7.81 to 9.06) | 2.43  (2.16 to 2.74) | 6.33  (5.96 to 6.73) | 1.74  (1.57 to 1.94) | 292.75  (275.43 to 311.64) | 81.95  (73.63 to 91.27) |
| **Female** | | | | | | |
| Australia | 3.82  (3.32 to 4.33) | 2.68  (2.18 to 3.27) | 2.41  (2.12 to 2.7) | 1.33  (1.14 to 1.58) | 112.41  (98.76 to 125.93) | 63.22  (53.94 to 75.14) |
| Brazil | 1.98  (1.77 to 2.23) | 2  (1.78 to 2.22) | 1.64  (1.47 to 1.86) | 1.6  (1.43 to 1.78) | 79.31  (70.72 to 89.96) | 77.84  (69.34 to 86.71) |
| China | 6.5  (4.59 to 8.48) | 3.63  (2.63 to 4.96) | 5.36  (3.8 to 7) | 2.61  (1.89 to 3.53) | 264.29  (185.92 to 345.67) | 125.29  (91.07 to 168.95) |
| Germany | 3.54  (3.17 to 3.95) | 3.39  (2.82 to 4.04) | 2.64  (2.39 to 2.9) | 2.03  (1.73 to 2.38) | 124.47  (112.19 to 137.19) | 94.68  (80.57 to 111.62) |
| India | 0.69  (0.47 to 0.98) | 1.11  (0.71 to 1.62) | 0.58  (0.39 to 0.82) | 0.91  (0.59 to 1.33) | 28.64  (19.34 to 40.7) | 44.06  (28.32 to 64.44) |
| Nigeria | 0.97  (0.61 to 1.47) | 1.18  (0.71 to 1.81) | 0.8  (0.51 to 1.22) | 0.97  (0.59 to 1.5) | 40.98  (25.99 to 62.72) | 48.66  (29.36 to 74.97) |
| Russia | 2.13  (1.78 to 2.58) | 1.58  (1.31 to 1.89) | 1.7  (1.42 to 2.07) | 1.14  (0.94 to 1.34) | 81.94  (68.4 to 99.62) | 54.98  (45.35 to 65.21) |
| USA | 6.63  (6 to 7.28) | 2.34  (1.99 to 2.8) | 4.77  (4.34 to 5.19) | 1.59  (1.35 to 1.86) | 220.94  (201.05 to 240.97) | 74.7  (63.61 to 88.35) |
| **Male** | | | | | | |
| Australia | 6.62  (5.88 to 7.45) | 3.34  (2.74 to 4.04) | 4.28  (3.84 to 4.77) | 1.69  (1.45 to 2.01) | 196.97  (176.3 to 220.04) | 79.45  (67.74 to 94.04) |
| Brazil | 4.25  (3.87 to 4.67) | 2.32  (2.08 to 2.58) | 3.57  (3.26 to 3.93) | 1.89  (1.7 to 2.11) | 168.31  (153.56 to 185.89) | 91.82  (82.43 to 102.46) |
| China | 8.6  (6.35 to 11.65) | 5.9  (4.09 to 8.05) | 7.2  (5.31 to 9.76) | 4.7  (3.26 to 6.41) | 349.48  (257.18 to 474.11) | 223.28  (155.03 to 304.47) |
| Germany | 11  (10.12 to 11.98) | 4.12  (3.49 to 4.92) | 8.73  (8.02 to 9.48) | 2.85  (2.44 to 3.31) | 402.03  (368.36 to 437.05) | 132.43  (113.33 to 154.39) |
| India | 1.82  (1.21 to 2.8) | 2.67  (1.7 to 3.99) | 1.54  (1.02 to 2.37) | 2.22  (1.41 to 3.32) | 73.37  (48.56 to 112.66) | 106.63  (67.57 to 159.37) |
| Nigeria | 1.64  (0.9 to 2.65) | 1.53  (0.87 to 2.52) | 1.39  (0.76 to 2.24) | 1.29  (0.73 to 2.12) | 66.31  (36.32 to 107.07) | 61.16  (34.69 to 100.7) |
| Russia | 15.83  (14.23 to 17.8) | 4.75  (4.2 to 5.45) | 13.32  (11.98 to 14.96) | 3.87  (3.42 to 4.43) | 611.07  (547.93 to 688.47) | 179.62  (158.68 to 206.26) |
| USA | 10.24  (9.44 to 11.26) | 2.51  (2.17 to 2.97) | 7.95  (7.29 to 8.68) | 1.9  (1.64 to 2.23) | 366.72  (335.45 to 401.25) | 89.12  (76.75 to 104.71) |

Note: AAPC, average annual percent change; ASR, age-standardised rate; CI, confidence interval; DALYs, disability-adjusted life years; EOTBL, early-onset Tracheal, bronchus, and lung; UI, uncertainty interval. The eight countries include Australia, Brazil, China, Germany, India, Nigeria, Russian, and USA.

**Table S7.** **Joinpoint regression analysis of EOTBL cancer incidence for both sexes across eight major countries from 1990 to 2023.**

|  | **Segment** | **Period** | **APC (95% CI)** | **P value** |
| --- | --- | --- | --- | --- |
| Australia | 1 | 1990-1994 | -3.10 (-6.42 to -1.23) | < 0.001 |
|  | 2 | 1994-2009 | 0.21 (-0.11 to 0.85) | 0.192 |
|  | 3 | 2009-2023 | -3.27 (-3.67 to -2.91) | < 0.001 |
| Brazil | 1 | 1990-2004 | -0.16 (-0.28 to -0.01) | 0.041 |
|  | 2 | 2004-2012 | -1.77 (-2.10 to -1.39) | 0.001 |
|  | 3 | 2012-2020 | -2.91 (-3.64 to -2.66) | < 0.001 |
|  | 4 | 2020-2023 | 1.70 (0.49 to 3.61) | 0.016 |
| China | 1 | 1990-2001 | 0.52 (0.35 to 0.75) | < 0.001 |
|  | 2 | 2001-2006 | -1.38 (-2.11 to -0.87) | < 0.001 |
|  | 3 | 2006-2012 | -5.17 (-5.63 to -4.84) | < 0.001 |
|  | 4 | 2012-2020 | -2.77 (-2.97 to -2.54) | < 0.001 |
|  | 5 | 2020-2023 | 3.45 (2.69 to 4.15) | < 0.001 |
| Germany | 1 | 1990-1995 | -1.69 (-2.96 to -1.05) | 0.009 |
|  | 2 | 1995-2000 | 0.77 (-0.26 to 2.11) | 0.077 |
|  | 3 | 2000-2008 | -1.45 (-2.10 to 0.56) | 0.053 |
|  | 4 | 2008-2011 | -5.61 (-6.20 to -0.97) | 0.004 |
|  | 5 | 2011-2019 | -3.26 (-5.46 to -2.78) | < 0.001 |
|  | 6 | 2019-2023 | -1.48 (-2.84 to 1.04) | 0.166 |
| India | 1 | 1990-2009 | 0.57 (-0.31 to 0.95) | 0.095 |
|  | 2 | 2009-2023 | 2.00 (1.47 to 3.25) | < 0.001 |
| Nigeria | 1 | 1990-1995 | -4.29 (-5.64 to -3.40) | < 0.001 |
|  | 2 | 1995-2011 | -1.03 (-1.20 to -0.85) | < 0.001 |
|  | 3 | 2011-2015 | 4.48 (3.21 to 6.19) | < 0.001 |
|  | 4 | 2015-2020 | -1.02 (-2.11 to -0.25) | 0.01 |
|  | 5 | 2020-2023 | 8.20 (6.48 to 10.04) | < 0.001 |
| Russia | 1 | 1990-1994 | 2.47 (1.28 to 4.51) | 0.003 |
|  | 2 | 1994-1997 | -7.50 (-9.03 to -4.76) | < 0.001 |
|  | 3 | 1997-2000 | -1.15 (-3.43 to 0.61) | 0.142 |
|  | 4 | 2000-2008 | -4.18 (-6.61 to -1.87) | 0.009 |
|  | 5 | 2008-2013 | -1.25 (-4.09 to 0.85) | 0.203 |
|  | 6 | 2013-2023 | -4.09 (-4.74 to -3.53) | 0.002 |
| USA | 1 | 1990-1997 | -2.68 (-3.11 to -2.34) | < 0.001 |
|  | 2 | 1997-2003 | -0.37 (-0.85 to 0.48) | 0.22 |
|  | 3 | 2003-2009 | -4.14 (-4.73 to -3.46) | < 0.001 |
|  | 4 | 2009-2018 | -6.99 (-7.47 to -6.67) | < 0.001 |
|  | 5 | 2018-2023 | -2.34 (-3.25 to -1.08) | 0.002 |

Note: EOTBL, early-onset Tracheal, bronchus, and lung; APC, annual percent change; CI, confidence interval.

**Table S8.** **Joinpoint regression analysis of EOTBL cancer mortality for both sexes across eight major countries from 1990 to 2023.**

|  | **Segment** | **Period** | **APC (95% CI)** | **P value** |
| --- | --- | --- | --- | --- |
| Australia | 1 | 1990-1997 | -2.90 (-5.56 to 1.86) | < 0.001 |
|  | 2 | 1997-2009 | -0.73 (-1.18 to 1.13) | 0.172 |
|  | 3 | 2009-2023 | -3.49 (-3.95 to -3.11) | < 0.001 |
| Brazil | 1 | 1990-2004 | -0.22 (-0.34 to -0.06) | 0.01 |
|  | 2 | 2004-2014 | -1.99 (-2.20 to -1.73) | 0.001 |
|  | 3 | 2014-2020 | -3.29 (-4.31 to -2.90) | < 0.001 |
|  | 4 | 2020-2023 | 1.58 (0.37 to 3.37) | 0.018 |
| China | 1 | 1990-2001 | 0.43 (0.25 to 0.66) | < 0.001 |
|  | 2 | 2001-2006 | -1.64 (-2.40 to -1.13) | < 0.001 |
|  | 3 | 2006-2012 | -5.46 (-5.92 to -5.12) | < 0.001 |
|  | 4 | 2012-2020 | -3.10 (-3.30 to -2.87) | < 0.001 |
|  | 5 | 2020-2023 | 3.06 (2.28 to 3.79) | < 0.001 |
| Germany | 1 | 1990-1995 | -2.76 (-4.46 to -1.98) | 0.001 |
|  | 2 | 1995-2003 | -0.84 (-1.23 to 0.49) | 0.133 |
|  | 3 | 2003-2008 | -3.20 (-4.19 to -2.04) | 0.001 |
|  | 4 | 2008-2011 | -6.33 (-7.05 to -4.62) | < 0.001 |
|  | 5 | 2011-2023 | -2.62 (-2.89 to -2.26) | 0.002 |
| India | 1 | 1990-2009 | 0.56 (-0.28 to 0.92) | 0.093 |
|  | 2 | 2009-2023 | 1.92 (1.40 to 3.07) | < 0.001 |
| Nigeria | 1 | 1990-1995 | -4.34 (-6.84 to -3.21) | < 0.001 |
|  | 2 | 1995-2011 | -1.04 (-1.27 to -0.79) | < 0.001 |
|  | 3 | 2011-2015 | 4.38 (2.92 to 6.33) | < 0.001 |
|  | 4 | 2015-2020 | -0.90 (-3.02 to -0.02) | 0.047 |
|  | 5 | 2020-2023 | 7.68 (5.28 to 11.69) | < 0.001 |
| Russia | 1 | 1990-1994 | 2.65 (1.41 to 4.83) | 0.002 |
|  | 2 | 1994-1997 | -7.65 (-9.21 to -4.84) | < 0.001 |
|  | 3 | 1997-2000 | -1.11 (-3.30 to 0.67) | 0.165 |
|  | 4 | 2000-2008 | -4.40 (-6.82 to -3.81) | 0.003 |
|  | 5 | 2008-2013 | -1.59 (-3.15 to 0.51) | 0.138 |
|  | 6 | 2013-2023 | -4.35 (-4.99 to -3.86) | < 0.001 |
| USA | 1 | 1990-1998 | -2.91 (-3.26 to -2.61) | < 0.001 |
|  | 2 | 1998-2003 | -0.34 (-1.02 to 0.80) | 0.35 |
|  | 3 | 2003-2009 | -4.34 (-4.92 to -3.68) | < 0.001 |
|  | 4 | 2009-2018 | -6.84 (-7.34 to -6.52) | < 0.001 |
|  | 5 | 2018-2023 | -2.52 (-3.50 to -1.06) | 0.006 |

Note: EOTBL, early-onset Tracheal, bronchus, and lung; APC, annual percent change; CI, confidence interval.

**Table S9. Joinpoint regression analysis of EOTBL cancer incidence for female across eight major countries from 1990 to 2023.**

|  | **Segment** | **Period** | **APC (95% CI)** | **P value** |
| --- | --- | --- | --- | --- |
| Australia | 1 | 1990-1992 | -4.24 (-6.14 to -0.94) | 0.012 |
|  | 2 | 1992-2009 | 1.28 (1.12 to 1.56) | < 0.001 |
|  | 3 | 2009-2012 | -5.46 (-6.63 to -3.21) | 0.019 |
|  | 4 | 2012-2015 | 1.44 (-0.95 to 3.02) | 0.213 |
|  | 5 | 2015-2020 | -6.35 (-8.68 to -5.41) | 0.005 |
|  | 6 | 2020-2023 | -0.42 (-2.99 to 3.82) | 0.779 |
| Brazil | 1 | 1990-1999 | 1.67 (1.47 to 2.06) | < 0.001 |
|  | 2 | 1999-2008 | 0.80 (0.47 to 1.05) | 0.006 |
|  | 3 | 2008-2016 | -1.37 (-1.62 to -1.09) | < 0.001 |
|  | 4 | 2016-2020 | -3.54 (-4.23 to -2.98) | < 0.001 |
|  | 5 | 2020-2023 | 1.90 (1.07 to 2.82) | < 0.001 |
| China | 1 | 1990-2001 | -0.45 (-0.61 to -0.26) | < 0.001 |
|  | 2 | 2001-2007 | -2.99 (-3.37 to -2.58) | < 0.001 |
|  | 3 | 2007-2013 | -5.22 (-5.53 to -4.97) | < 0.001 |
|  | 4 | 2013-2020 | -2.00 (-2.16 to -1.81) | < 0.001 |
|  | 5 | 2020-2023 | 3.95 (3.23 to 4.57) | < 0.001 |
| Germany | 1 | 1990-1992 | 6.36 (3.45 to 8.31) | < 0.001 |
|  | 2 | 1992-2002 | 3.01 (0.63 to 3.30) | 0.024 |
|  | 3 | 2002-2008 | 0.19 (-1.59 to 1.04) | 0.577 |
|  | 4 | 2008-2014 | -3.94 (-5.24 to -2.96) | 0.01 |
|  | 5 | 2014-2023 | -2.58 (-3.08 to -1.23) | 0.015 |
| India | 1 | 1990-2001 | 1.14 (0.87 to 1.49) | 0.011 |
|  | 2 | 2001-2004 | -2.26 (-2.96 to -0.52) | 0.034 |
|  | 3 | 2004-2011 | 0.27 (-0.34 to 1.73) | 0.17 |
|  | 4 | 2011-2016 | 5.15 (4.32 to 6.54) | < 0.001 |
|  | 5 | 2016-2020 | -0.44 (-1.81 to 0.58) | 0.268 |
|  | 6 | 2020-2023 | 6.80 (5.23 to 9.79) | < 0.001 |
| Nigeria | 1 | 1990-1996 | -3.37 (-4.40 to -2.69) | < 0.001 |
|  | 2 | 1996-2008 | -0.84 (-2.28 to -0.53) | 0.013 |
|  | 3 | 2008-2012 | 1.56 (-0.75 to 2.73) | 0.172 |
|  | 4 | 2012-2015 | 5.98 (3.96 to 6.98) | 0.004 |
|  | 5 | 2015-2020 | -0.59 (-1.41 to 0.01) | 0.05 |
|  | 6 | 2020-2023 | 10.05 (8.67 to 11.34) | < 0.001 |
| Russia | 1 | 1990-1994 | 3.06 (1.89 to 5.14) | < 0.001 |
|  | 2 | 1994-1997 | -4.04 (-5.00 to -2.00) | < 0.001 |
|  | 3 | 1997-2007 | -1.40 (-1.75 to 0.21) | 0.054 |
|  | 4 | 2007-2011 | 2.43 (0.54 to 3.99) | 0.045 |
|  | 5 | 2011-2020 | -0.87 (-1.40 to -0.26) | 0.033 |
|  | 6 | 2020-2023 | -5.42 (-8.04 to -3.79) | < 0.001 |
| USA | 1 | 1990-1997 | -1.69 (-2.20 to -1.32) | 0.006 |
|  | 2 | 1997-2002 | 1.29 (-1.37 to 2.24) | 0.106 |
|  | 3 | 2002-2005 | -1.30 (-2.86 to 1.23) | 0.168 |
|  | 4 | 2005-2009 | -3.26 (-7.46 to -2.69) | < 0.001 |
|  | 5 | 2009-2019 | -6.99 (-7.42 to -6.28) | < 0.001 |
|  | 6 | 2019-2023 | -2.21 (-3.31 to -0.17) | 0.038 |

Note: EOTBL, early-onset Tracheal, bronchus, and lung; APC, annual percent change; CI, confidence interval.

**Table S10.** **Joinpoint regression analysis of EOTBL cancer mortality for female across eight major countries from 1990 to 2023.**

|  | **Segment** | **Period** | **APC (95% CI)** | **P value** |
| --- | --- | --- | --- | --- |
| Australia | 1 | 1990-1992 | -4.92 (-6.94 to -1.14) | 0.001 |
|  | 2 | 1992-2009 | 0.24 (0.08 to 0.58) | 0.009 |
|  | 3 | 2009-2012 | -6.30 (-7.47 to -4.17) | 0.007 |
|  | 4 | 2012-2015 | 1.18 (-1.09 to 2.70) | 0.256 |
|  | 5 | 2015-2020 | -6.52 (-8.68 to -5.68) | 0.004 |
|  | 6 | 2020-2023 | -0.79 (-3.32 to 3.45) | 0.559 |
| Brazil | 1 | 1990-1999 | 1.63 (1.41 to 2.05) | < 0.001 |
|  | 2 | 1999-2008 | 0.72 (0.35 to 0.97) | 0.007 |
|  | 3 | 2008-2016 | -1.46 (-1.73 to -1.16) | < 0.001 |
|  | 4 | 2016-2020 | -3.65 (-4.48 to -3.07) | < 0.001 |
|  | 5 | 2020-2023 | 1.47 (0.54 to 2.74) | 0.006 |
| China | 1 | 1990-2001 | -0.59 (-0.73 to -0.42) | < 0.001 |
|  | 2 | 2001-2007 | -3.45 (-3.79 to -3.04) | < 0.001 |
|  | 3 | 2007-2013 | -5.82 (-6.08 to -5.61) | < 0.001 |
|  | 4 | 2013-2020 | -2.55 (-2.70 to -2.39) | < 0.001 |
|  | 5 | 2020-2023 | 3.43 (2.80 to 4.02) | < 0.001 |
| Germany | 1 | 1990-2002 | 2.01 (1.72 to 2.39) | 0.001 |
|  | 2 | 2002-2008 | -0.97 (-1.89 to 0.11) | 0.065 |
|  | 3 | 2008-2013 | -4.36 (-5.91 to -3.44) | 0.001 |
|  | 4 | 2013-2023 | -2.50 (-2.91 to -1.37) | 0.01 |
| India | 1 | 1990-2001 | 1.03 (0.77 to 1.36) | 0.01 |
|  | 2 | 2001-2004 | -2.08 (-2.76 to -0.48) | 0.038 |
|  | 3 | 2004-2011 | 0.19 (-0.31 to 1.36) | 0.287 |
|  | 4 | 2011-2015 | 5.79 (4.60 to 7.21) | 0.004 |
|  | 5 | 2015-2020 | 0.20 (-0.85 to 0.96) | 0.567 |
|  | 6 | 2020-2023 | 6.32 (4.71 to 8.87) | < 0.001 |
| Nigeria | 1 | 1990-1996 | -3.28 (-4.14 to -2.65) | < 0.001 |
|  | 2 | 1996-2010 | -0.82 (-1.03 to -0.59) | < 0.001 |
|  | 3 | 2010-2015 | 4.89 (3.95 to 6.40) | < 0.001 |
|  | 4 | 2015-2020 | -0.41 (-1.45 to 0.41) | 0.239 |
|  | 5 | 2020-2023 | 9.66 (8.14 to 11.12) | < 0.001 |
| Russia | 1 | 1990-1993 | 3.85 (1.41 to 8.15) | 0.002 |
|  | 2 | 1993-2007 | -2.01 (-2.39 to -1.76) | < 0.001 |
|  | 3 | 2007-2011 | 2.03 (0.37 to 3.88) | 0.018 |
|  | 4 | 2011-2020 | -1.38 (-2.00 to -0.80) | 0.007 |
|  | 5 | 2020-2023 | -5.63 (-8.80 to -3.82) | < 0.001 |
| USA | 1 | 1990-1998 | -2.00 (-2.49 to -1.65) | 0.011 |
|  | 2 | 1998-2002 | 1.38 (-1.90 to 2.35) | 0.107 |
|  | 3 | 2002-2006 | -1.73 (-2.66 to 1.41) | 0.108 |
|  | 4 | 2006-2009 | -3.73 (-7.17 to -2.22) | < 0.001 |
|  | 5 | 2009-2019 | -6.82 (-7.56 to -6.30) | < 0.001 |
|  | 6 | 2019-2023 | -2.51 (-3.85 to 0.09) | 0.054 |

Note: EOTBL, early-onset Tracheal, bronchus, and lung; APC, annual percent change; CI, confidence interval.

**Table S11. Joinpoint regression analysis of EOTBL cancer incidence for male across eight major countries from 1990 to 2023.**

|  | **Segment** | **Period** | **APC (95% CI)** | **P value** |
| --- | --- | --- | --- | --- |
| Australia | 1 | 1990-1996 | -2.89 (-5.77 to -0.20) | 0.046 |
|  | 2 | 1996-2005 | -0.81 (-4.30 to 2.06) | 0.331 |
|  | 3 | 2005-2008 | 1.94 (-5.25 to 3.31) | 0.697 |
|  | 4 | 2008-2023 | -3.28 (-7.24 to -0.66) | 0.03 |
| Brazil | 1 | 1990-2004 | -1.05 (-1.22 to -0.85) | < 0.001 |
|  | 2 | 2004-2020 | -3.12 (-3.31 to -2.97) | < 0.001 |
|  | 3 | 2020-2023 | 2.05 (0.34 to 4.84) | 0.026 |
| China | 1 | 1990-2001 | 1.20 (0.96 to 1.48) | 0.004 |
|  | 2 | 2001-2006 | -0.57 (-1.40 to 1.14) | 0.104 |
|  | 3 | 2006-2012 | -5.19 (-5.70 to -0.69) | 0.001 |
|  | 4 | 2012-2017 | -2.59 (-5.21 to -1.69) | < 0.001 |
|  | 5 | 2017-2020 | -3.96 (-4.49 to -3.11) | < 0.001 |
|  | 6 | 2020-2023 | 3.70 (2.74 to 4.49) | < 0.001 |
| Germany | 1 | 1990-1994 | -3.88 (-6.44 to -2.46) | < 0.001 |
|  | 2 | 1994-2003 | -1.32 (-1.85 to 0.28) | 0.078 |
|  | 3 | 2003-2017 | -4.17 (-4.85 to -3.86) | 0.003 |
|  | 4 | 2017-2023 | -1.74 (-3.13 to 1.53) | 0.156 |
| India | 1 | 1990-2019 | 1.06 (-0.13 to 1.29) | 0.055 |
|  | 2 | 2019-2023 | 4.85 (1.29 to 11.72) | < 0.001 |
| Nigeria | 1 | 1990-1995 | -4.44 (-6.65 to -3.33) | < 0.001 |
|  | 2 | 1995-2011 | -0.77 (-1.01 to -0.52) | < 0.001 |
|  | 3 | 2011-2015 | 3.65 (2.20 to 5.49) | 0.002 |
|  | 4 | 2015-2020 | -1.15 (-3.34 to -0.25) | 0.014 |
|  | 5 | 2020-2023 | 6.62 (4.22 to 10.53) | < 0.001 |
| Russia | 1 | 1990-1994 | 0.51 (-2.43 to 6.60) | 0.733 |
|  | 2 | 1994-2023 | -3.95 (-4.16 to -3.79) | < 0.001 |
| USA | 1 | 1990-1998 | -3.22 (-3.97 to -2.84) | < 0.001 |
|  | 2 | 1998-2004 | -1.55 (-2.25 to 0.03) | 0.052 |
|  | 3 | 2004-2018 | -6.44 (-6.73 to -6.22) | < 0.001 |
|  | 4 | 2018-2023 | -1.91 (-3.22 to 0.07) | 0.058 |

Note: EOTBL, early-onset Tracheal, bronchus, and lung; APC, annual percent change; CI, confidence interval.

**Table S12. Joinpoint regression analysis of EOTBL cancer mortality for male across eight major countries from 1990 to 2023.**

|  | **Segment** | **Period** | **APC (95% CI)** | **P value** |
| --- | --- | --- | --- | --- |
| Australia | 1 | 1990-1997 | -4.25 (-7.56 to -3.03) | < 0.001 |
|  | 2 | 1997-2009 | -1.42 (-1.95 to 2.24) | 0.188 |
|  | 3 | 2009-2023 | -3.21 (-3.96 to -2.77) | 0.001 |
| Brazil | 1 | 1990-2004 | -1.11 (-1.27 to -0.91) | < 0.001 |
|  | 2 | 2004-2020 | -3.22 (-3.40 to -3.08) | < 0.001 |
|  | 3 | 2020-2023 | 1.89 (0.19 to 4.74) | 0.028 |
| China | 1 | 1990-2001 | 1.13 (0.92 to 1.39) | 0.003 |
|  | 2 | 2001-2006 | -0.74 (-1.54 to 0.29) | 0.06 |
|  | 3 | 2006-2012 | -5.37 (-5.86 to -2.21) | 0.001 |
|  | 4 | 2012-2017 | -2.73 (-5.07 to -1.85) | < 0.001 |
|  | 5 | 2017-2020 | -4.19 (-4.71 to -3.34) | < 0.001 |
|  | 6 | 2020-2023 | 3.47 (2.52 to 4.23) | < 0.001 |
| Germany | 1 | 1990-1996 | -4.22 (-5.61 to -3.55) | < 0.001 |
|  | 2 | 1996-2000 | -0.95 (-2.27 to 0.58) | 0.237 |
|  | 3 | 2000-2008 | -3.76 (-4.39 to -3.22) | 0.001 |
|  | 4 | 2008-2011 | -7.62 (-8.45 to -5.56) | 0.002 |
|  | 5 | 2011-2023 | -2.48 (-2.83 to -2.03) | 0.002 |
| India | 1 | 1990-2019 | 1.02 (-0.77 to 1.72) | 0.069 |
|  | 2 | 2019-2023 | 4.64 (1.12 to 11.74) | 0.011 |
| Nigeria | 1 | 1990-1995 | -4.38 (-6.67 to -3.28) | < 0.001 |
|  | 2 | 1995-2011 | -0.82 (-1.07 to -0.58) | 0.001 |
|  | 3 | 2011-2015 | 3.64 (2.22 to 5.46) | 0.002 |
|  | 4 | 2015-2020 | -1.11 (-3.33 to -0.26) | 0.016 |
|  | 5 | 2020-2023 | 6.47 (4.03 to 10.45) | < 0.001 |
| Russia | 1 | 1990-1994 | 0.78 (-2.20 to 6.62) | 0.612 |
|  | 2 | 1994-2023 | -4.09 (-4.29 to -3.92) | < 0.001 |
| USA | 1 | 1990-1998 | -3.47 (-4.05 to -3.11) | < 0.001 |
|  | 2 | 1998-2004 | -1.69 (-2.33 to -0.24) | 0.034 |
|  | 3 | 2004-2018 | -6.40 (-6.66 to -6.20) | < 0.001 |
|  | 4 | 2018-2023 | -2.00 (-3.24 to -0.14) | 0.04 |

Note: EOTBL, early-onset Tracheal, bronchus, and lung; APC, annual percent change; CI, confidence interval.

**Table S13. Percentage contribution of the remaining 12 risk factors to DALYs of EOTBL cancer by sex across the eight**

**main countries in 2023.**

| **Location** | **Risk factors** | | | | | | | | | | | |
| --- | --- | --- | --- | --- | --- | --- | --- | --- | --- | --- | --- | --- |
|  | Diet low in fruits, % (95% UI) | High fasting plasma glucose, % (95% UI) | Occupational exposure to arsenic,  % (95%  UI) | Occupational exposure to asbestos,  % (95%  UI) | Occupational exposure to beryllium,  % (95%  UI) | Occupational exposure to cadmium,  % (95%  UI) | Occupational exposure to chromium,  % (95%  UI) | Occupational exposure to diesel engine exhaust, % (95% UI) | Occupational exposure to nickel,  % (95%  UI) | Occupational exposure to polycyclic aromatic hydrocarbons, % (95% UI) | Occupational exposure to silica, % (95% UI) | Residential radon, % (95% UI) |
| **Both** | | | | | | | | | | | | |
| Australia | 3.5  (1.7-5.9) | 3.0  (1.4-6.1) | 0.6  (-0.1-1.4) | 1.3  (0.7-2.0) | 0.0  (0.0-0.0) | 0.0  (0.0-0.0) | 0.0  (0.0-0.0) | 0.6  (0.5-0.8) | 0.5  (-0.2-2.0) | 0.1  (0.1-0.2) | 4.1  (1.1-7.0) | 1.2  (-0.5-4.2) |
| Brazil | 2.6  (1.3-4.4) | 3.8  (1.8-7.4) | 0.6  (0.2-1.0) | 1.5  (1.0-2.2) | 0.0  (0.0-0.0) | 0.1  (0.0-0.1) | 0.2  (0.1-0.2) | 2.3  (2.0-2.8) | 0.7  (0.1-1.4) | 0.6  (0.4-0.7) | 3.9  (1.8-5.9) | 4.1  (-1.8-11.9) |
| China | 5.0  (2.4-8.2) | 3.3  (1.6-5.6) | 1.1  (0.4-1.7) | 0.6  (0.3-1.0) | 0.0  (0.0-0.0) | 0.1  (0.1-0.1) | 0.2  (0.2-0.3) | 2.3  (2.0-2.9) | 1.0  (0.2-2.2) | 0.7  (0.6-0.9) | 4.7  (2.2-7.1) | 3.7  (-1.6-9.5) |
| Germany | 2.7  (1.3-4.5) | 3.0  (1.6-4.7) | 0.9  (-0.2-1.8) | 0.7  (0.4-1.2) | 0.0  (0.0-0.0) | 0.0  (0.0-0.0) | 0.0  (0.0-0.1) | 0.5  (0.5-0.7) | 0.7  (-0.3-2.5) | 0.2  (0.1-0.2) | 4.3  (1.2-7.2) | 5.4  (-2.3-16.1) |
| India | 19.4  (10.3-27.5) | 2.9  (1.4-5.4) | 0.6  (0.2-1.0) | 0.4  (0.2-0.7) | 0.0  (0.0-0.0) | 0.1  (0.0-0.1) | 0.1  (0.1-0.1) | 2.1  (1.7-2.6) | 0.7  (0.2-1.6) | 0.5  (0.3-0.6) | 5.2  (2.5-8.0) | 4.4  (-1.6-12.0) |
| Nigeria | 5.5  (2.4-10.9) | 1.9  (0.8-3.9) | 0.4  (0.2-0.7) | 0.1  (0.0-0.1) | 0.0  (0.0-0.0) | 0.0  (0.0-0.1) | 0.1  (0.1-0.1) | 2.0  (1.7-2.4) | 0.5  (0.1-1.2) | 0.4  (0.3-0.5) | 3.6  (1.7-5.6) | 4.5  (-2.4-11.6) |
| Russia | 5.1  (2.4-8.7) | 2.0  (0.9-4.0) | 0.6  (-0.1-1.3) | 1.1  (0.6-1.8) | 0.0  (0.0-0.0) | 0.0  (0.0-0.0) | 0.0  (0.0-0.0) | 0.5  (0.4-0.6) | 0.5  (-0.2-2.0) | 0.1  (0.1-0.1) | 3.9  (1.0-6.6) | 7.4  (-3.1-19.2) |
| USA | 2.9  (1.4-4.8) | 3.5  (1.7-6.5) | 0.7  (-0.2-1.5) | 0.3  (0.2-0.5) | 0.0  (0.0-0.0) | 0.0  (0.0-0.0) | 0.0  (0.0-0.0) | 0.5  (0.4-0.6) | 0.5  (-0.2-1.8) | 0.1  (0.1-0.2) | 2.7  (0.7-4.5) | 4.5  (-2.1-12.1) |
| **Female** | | | | | | | | | | | | |
| Australia | 3.0  (1.5-4.9) | 2.8  (1.3-5.8) | 0.6  (-0.1-1.3) | 0.5  (0.3-0.9) | 0.0  (0.0-0.0) | 0.0  (0.0-0.0) | 0.0  (0.0-0.0) | 0.6  (0.4-0.7) | 0.4  (-0.2-1.5) | 0.1  (0.1-0.2) | 2.5  (0.6-4.2) | 1.2  (-0.5-4.2) |
| Brazil | 2.6  (1.3-4.6) | 4.3  (1.9-8.1) | 0.6  (0.2-1.0) | 0.7  (0.5-1.0) | 0.0  (0.0-0.0) | 0.1  (0.0-0.1) | 0.2  (0.1-0.2) | 1.9  (1.4-2.3) | 0.5  (0.1-1.1) | 0.6  (0.4-0.7) | 2.1  (0.9-3.2) | 4.1  (-1.8-11.8) |
| China | 4.1  (2.0-6.6) | 3.0  (1.5-5.2) | 1.2  (0.5-2.0) | 0.2  (0.1-0.4) | 0.0  (0.0-0.0) | 0.1  (0.1-0.2) | 0.3  (0.2-0.3) | 2.5  (1.8-3.1) | 1.1  (0.2-2.3) | 0.9  (0.6-1.2) | 4.6  (2.1-7.0) | 3.8  (-1.6-9.8) |
| Germany | 2.3  (1.1-3.8) | 2.6  (1.2-5.3) | 0.8  (-0.1-1.7) | 0.2  (0.1-0.4) | 0.0  (0.0-0.0) | 0.0  (0.0-0.0) | 0.0  (0.0-0.1) | 0.5  (0.4-0.7) | 0.5  (-0.2-2.0) | 0.2  (0.1-0.2) | 2.9  (0.7-4.9) | 5.4  (-2.3-16.1) |
| India | 19.4  (10.1-27.5) | 3.1  (1.4-6.1) | 0.3  (0.1-0.4) | 0.0  (0.0-0.1) | 0.0  (0.0-0.0) | 0.0  (0.0-0.0) | 0.1  (0.0-0.1) | 0.7  (0.5-0.8) | 0.3  (0.1-0.7) | 0.2  (0.1-0.3) | 1.9  (0.9-3.1) | 4.5  (-1.6-11.9) |
| Nigeria | 5.5  (2.4-10.9) | 1.8  (0.7-3.9) | 0.5  (0.2-0.7) | 0.0  (0.0-0.0) | 0.0  (0.0-0.0) | 0.0  (0.0-0.1) | 0.1  (0.1-0.1) | 1.8  (1.4-2.2) | 0.5  (0.1-1.1) | 0.4  (0.3-0.5) | 2.6  (1.2-4.0) | 4.5  (-2.3-11.5) |
| Russia | 4.0  (1.9-6.9) | 2.3  (1.1-5.0) | 0.7  (-0.1-1.5) | 0.5  (0.3-0.8) | 0.0  (0.0-0.0) | 0.0  (0.0-0.0) | 0.0  (0.0-0.0) | 0.5  (0.4-0.6) | 0.5  (-0.2-1.9) | 0.1  (0.1-0.2) | 2.9  (0.7-4.8) | 7.5  (-3.2-19.2) |
| USA | 2.5  (1.2-4.2) | 3.4  (1.6-6.5) | 0.6  (-0.1-1.3) | 0.2  (0.1-0.3) | 0.0  (0.0-0.0) | 0.0  (0.0-0.0) | 0.0  (0.0-0.0) | 0.5  (0.4-0.7) | 0.4  (-0.2-1.6) | 0.1  (0.1-0.2) | 2.1  (0.6-3.7) | 4.6  (-2.1-12.1) |
| **Male** | | | | | | | | | | | | |
| Australia | 3.9  (1.9-6.6) | 3.3  (1.5-6.4) | 0.7  (-0.1-1.4) | 1.9  (1.0-3.1) | 0.0  (0.0-0.0) | 0.0  (0.0-0.0) | 0.0  (0.0-0.0) | 0.7  (0.5-0.9) | 0.6  (-0.3-2.3) | 0.1  (0.1-0.2) | 5.4  (1.4-9.1) | 1.2  (-0.5-4.2) |
| Brazil | 2.5  (1.2-4.3) | 3.4  (1.6-6.7) | 0.7  (0.3-1.1) | 2.2  (1.3-3.4) | 0.0  (0.0-0.0) | 0.1  (0.0-0.1) | 0.2  (0.1-0.2) | 2.8  (2.3-3.4) | 0.8  (0.2-1.8) | 0.6  (0.4-0.7) | 5.5  (2.6-8.2) | 4.1  (-1.8-11.9) |
| China | 5.5  (2.7-9.2) | 3.4  (1.7-5.8) | 1.0  (0.4-1.6) | 0.8  (0.4-1.5) | 0.0  (0.0-0.0) | 0.0  (0.0-0.1) | 0.2  (0.2-0.3) | 2.3  (1.8-2.9) | 1.0  (0.2-2.1) | 0.7  (0.4-0.9) | 4.7  (2.2-7.3) | 3.7  (-1.6-9.5) |
| Germany | 3.1  (1.5-5.0) | 3.2  (1.5-6.0) | 0.9  (-0.2-2.0) | 1.0  (0.5-1.8) | 0.0  (0.0-0.0) | 0.0  (0.0-0.0) | 0.0  (0.0-0.1) | 0.6  (0.4-0.7) | 0.8  (-0.3-2.8) | 0.2  (0.1-0.2) | 5.3  (1.3-8.9) | 5.4  (-2.3-16.1) |
| India | 19.4  (10.3-27.6) | 2.8  (1.4-5.2) | 0.7  (0.3-1.1) | 0.6  (0.3-1.0) | 0.0  (0.0-0.0) | 0.0  (0.0-0.0) | 0.1  (0.1-0.2) | 2.7  (2.2-3.4) | 0.9  (0.2-1.9) | 0.6  (0.4-0.7) | 6.5  (3.0-10.0) | 4.4  (-1.6-12.1) |
| Nigeria | 5.4  (2.4-10.7) | 1.9  (0.9-3.9) | 0.4  (0.2-0.7) | 0.1  (0.1-0.2) | 0.0  (0.0-0.0) | 0.0  (0.0-0.1) | 0.1  (0.1-0.1) | 2.2  (1.8-2.8) | 0.6  (0.1-1.3) | 0.4  (0.3-0.5) | 4.6  (2.1-7.0) | 4.5  (-2.5-11.7) |
| Russia | 5.4  (2.5-9.2) | 1.9  (0.9-3.7) | 0.6  (-0.1-1.3) | 1.3  (0.7-2.2) | 0.0  (0.0-0.0) | 0.0  (0.0-0.0) | 0.0  (0.0-0.0) | 0.5  (0.4-0.6) | 0.5  (-0.2-2.0) | 0.1  (0.1-0.1) | 4.2  (1.1-7.3) | 7.4  (-3.1-19.1) |
| USA | 3.3  (1.6-5.5) | 3.6  (1.8-6.5) | 0.8  (-0.2-1.6) | 0.5  (0.3-0.8) | 0.0  (0.0-0.0) | 0.0  (0.0-0.0) | 0.0  (0.0-0.0) | 0.5  (0.4-0.7) | 0.6  (-0.2-2.1) | 0.1  (0.1-0.2) | 3.1  (0.8-5.3) | 4.5  (-2.1-12.2) |

Note：DALYs, disability-adjusted life years; EOTBL, early-onset Tracheal, bronchus, and lung; UI, uncertainty interval.
